# Scaling up quality care for mothers and newborns around the time of birth: an overview of methods and analyses of intervention-specific bottlenecks and solutions

**DOI:** 10.1186/1471-2393-15-S2-S1

**Published:** 2015-09-11

**Authors:** Kim E Dickson, Mary V Kinney, Sarah G Moxon, Joanne Ashton, Nabila Zaka, Aline Simen-Kapeu, Gaurav Sharma, Kate J Kerber, Bernadette Daelmans, A Metin Gülmezoglu, Matthews Mathai, Christabel Nyange, Martina Baye, Joy E Lawn

**Affiliations:** 1 Health Section, Programme Division, UNICEF Headquarters, 3 United Nations Plaza, New York, NY, 10017, USA; 2 Saving Newborn Lives, Save the Children, 2000 L Street NW, Suite 500, Washington, DC 20036, USA; 3 Maternal, Adolescent, Reproductive and Child Health (MARCH) Centre, London School of Hygiene and Tropical Medicine, London, WC1E 7HT, UK; 4 Department of Infectious Disease Epidemiology, London School of Hygiene and Tropical Medicine, London, WC1E 7HT, UK; 5 5919 N Placita del Conde, Tucson, Arizona 85718, USA; 6 Department of Maternal, Newborn, Child and Adolescent Health, World Health Organization, Avenue Appia 20, 1211 Geneva 27, Switzerland; 7 UNDP/UNFPA/UNICEF/WHO/World Bank Special Programme of Research, Development and Research Training in Human Reproduction (HRP), Department of Reproductive Health and Research, World Health Organization, 20 Avenue Appia, CH-1211, Geneva, Switzerland; 8 Ross University Medical School, 2300 SW 145th Avenue, Miramar, FL, 33027, USA; 9 National Program for Reduction of Maternal Newborn and Child Mortality, Ministry of Public Health Cameroon, Cameroon

**Keywords:** Quality of care, pregnancy, childbirth, health systems, bottlenecks, maternal, newborn, stillbirths

## Abstract

**Background:**

The Every Newborn Action Plan (ENAP) and Ending Preventable Maternal Mortality targets cannot be achieved without high quality, equitable coverage of interventions at and around the time of birth. This paper provides an overview of the methodology and findings of a nine paper series of in-depth analyses which focus on the specific challenges to scaling up high-impact interventions and improving quality of care for mothers and newborns around the time of birth, including babies born small and sick.

**Methods:**

The bottleneck analysis tool was applied in 12 countries in Africa and Asia as part of the ENAP process. Country workshops engaged technical experts to complete a tool designed to synthesise "bottlenecks" hindering the scale up of maternal-newborn intervention packages across seven health system building blocks. We used quantitative and qualitative methods and literature review to analyse the data and present priority actions relevant to different health system building blocks for skilled birth attendance, emergency obstetric care, antenatal corticosteroids (ACS), basic newborn care, kangaroo mother care (KMC), treatment of neonatal infections and inpatient care of small and sick newborns.

**Results:**

The 12 countries included in our analysis account for the majority of global maternal (48%) and newborn (58%) deaths and stillbirths (57%). Our findings confirm previously published results that the interventions with the most perceived bottlenecks are facility-based where rapid emergency care is needed, notably inpatient care of small and sick newborns, ACS, treatment of neonatal infections and KMC. Health systems building blocks with the highest rated bottlenecks varied for different interventions. Attention needs to be paid to the context specific bottlenecks for each intervention to scale up quality care. Crosscutting findings on health information gaps inform two final papers on a roadmap for improvement of coverage data for newborns and indicate the need for leadership for effective audit systems.

**Conclusions:**

Achieving the Sustainable Development Goal targets for ending preventable mortality and provision of universal health coverage will require large-scale approaches to improving quality of care. These analyses inform the development of systematic, targeted approaches to strengthening of health systems, with a focus on overcoming specific bottlenecks for the highest impact interventions.

## Background

Poor quality of maternal and newborn care during pregnancy, childbirth and in the postnatal period significantly contributes to the annual estimated 289,000 maternal deaths [1], 2.6 million stillbirths [2] and 2.8 million newborn deaths globally [3]. Women and newborns are at greatest risk at and around the time of birth, and babies born small and sick are especially vulnerable [4]. Available interventions can prevent many of these deaths [5], but interventions often face challenges to scale up, many of which are specific to context or the intervention [4]. Understanding these specific challenges is critical to aid countries to intentionally focus their efforts and resources to achieve the effective, high quality coverage of interventions that are needed to save women and newborns, and to prevent stillbirths.

In May 2014, the 67^th ^World Health Assembly endorsed the Every Newborn Action Plan (ENAP), which set a target of ≤12 neonatal deaths per 1000 live births and stillbirths per 1000 total births by 2030 and set eight specific milestones at global and country level to 2020 [6]. The ENAP impact framework [7], inserts *"Every Newborn" *into the "*Every Woman, Every Child*" concept, broadening its goals to include ending preventable stillbirths and deaths for women, newborns and children, and improving child development and human capital. Effective interventions for improving the survival and health of newborns forms one component of integrated health services for reproductive, maternal, newborn, child and adolescent health (RMNCAH). The identified core ENAP interventions are packaged for levels of service delivery and are delivered from common platforms. Ensuring equitable coverage of high quality health care for women and children, including care at the start of life, must be placed at the heart of the post-2015 Sustainable Development Framework. The ENAP together with the *Strategy for Ending Preventable Maternal Mortality *(EPMM) [8] provide a strong investment case for women's and children's health with clear actions and goals for maternal and newborn health post-2015 [6,8]. Achieving the targets also requires functioning health systems, integrated planning and delivery to ensure efficient, high quality and effective health services for women and children [4].

### Quality of care

The issue of quality of care remains central to maternal and newborn health since increasing coverage of interventions alone will not necessarily deliver the outcomes or impact needed to reach mortality reduction targets [9]. Stagnation in neonatal mortality rates (NMR) is being observed even in the context of rapid improvements in coverage of skilled birth attendance and facility-based births [10]. For example, in South Africa, more than 95% of births are facility-based, but NMR has hardly shifted in recent years, most probably due to inadequate quality of care during pregnancy, childbirth and the postnatal period [11]. Similarly, the evaluation of the conditional cash transfer Janani Suraksha Yojana program in India showed significant increases in facility deliveries but no change in NMR; the impact on maternal health outcomes was also unclear [12]. A recent analysis by Bhutta et al [5] modelled the effect and cost of scaling up available interventions for mothers and newborns at and around the time of birth; estimates suggest that improving the quality of care could have the greatest impact, resulting in a triple return on investment saving women, newborns, preventing stillbirths and could also prevent millions of babies from suffering disabilities related to insults at the time of birth.

Quality of care in itself is a difficult concept to define; traditionally, the concept of quality of medical care has been conceptualised as the provision of care according to defined standards that are affordable to the society in question, and have the ability to produce an impact on mortality, morbidity and disability [13]. Hulton and colleagues introduced the issue of reproductive rights and the importance of the dual concepts of the 'provision of care' and 'experience of care'; the latter emphasises the importance of the patient's perspective of the care received [14]. The Donabedian Model provides one of the earliest conceptual frameworks for examining health services and evaluating quality of care based on three categories: "structure," "process," and "outcomes" [15]. Structure describes the context in which care is delivered, including hospital buildings, staff, financing, and equipment. Process denotes the transactions between patients and providers throughout the delivery of healthcare. Finally, outcome refers to the effects of healthcare on the health status of patients and populations. Other frameworks build on this concept to make the measurement of quality more specific to maternal health services [14], and most recently to ensure the different levels of the health care system are considered [9]. Given the inextricable link between maternal and newborn health, the care received by a mother is critical in influencing her outcomes as well as the outcomes of her baby and frameworks for measuring quality of care provided for mothers should also consider outcomes for newborns. Van Lerberghe and colleagues [16] recently explored the diverse actions that have contributed to health system strengthening over the past 25 years in four settings; they found that attention for quality of care only really began when uptake of care had already substantially increased. To achieve quality even where scale up has been achieved, there are areas of difficulty or context specific challenges that need to be addressed. Alongside increasing availability and coverage of services, tackling the issue of quality has been identified as a moral and public health imperative.

### Health system bottlenecks to the provision of quality maternal and newborn care

This is the first paper in a supplement of nine papers that provides an overview of the methodology and findings of a new set of analyses using data from 12 high-burden countries to carry out an in-depth exploration of the intervention-specific bottlenecks to the scale up of quality care around the time of birth and for small and sick newborns [17-22]. The aim of this work is to use the bottleneck analysis as a systematic approach to identify challenges and implementable actions to scale up quality care. This paper presents the systematic approach that was used to conduct the in-depth analyses to identify and unpack the critical bottlenecks by health system building block for each of the nine high-impact interventions for care around the time of birth and for small and sick newborns. Health systems strengthening will only be accomplished by comprehensive changes to policies and regulations, organisational structures, and relationships across the health systems building blocks that motivate changes in behaviour, and/or allow more effective use of resources to improve multiple health services [23]. We therefore use the health systems building block as the basis to collect and report data in a way that can be applied to analyse specific challenges and identify practical solutions to improve the implementation of services and strengthen health systems.

A year after the launch of the ENAP, significant progress has been made to support and invest in maternal and newborn health, but further progress will only be made with attention to specific implementation challenges, many of which vary by context and intervention. The papers in this series build on the analyses and evidence published previously in *The Lancet *Every Newborn Series [4], expanding the analysis to include data from four additional countries (12 in total) and presenting a more in-depth analysis of the different challenges for each of the maternal-newborn intervention packages. The papers also discuss the policy and programmatic implications and priority actions for programme scale up for each intervention package.

**Figure 1:**
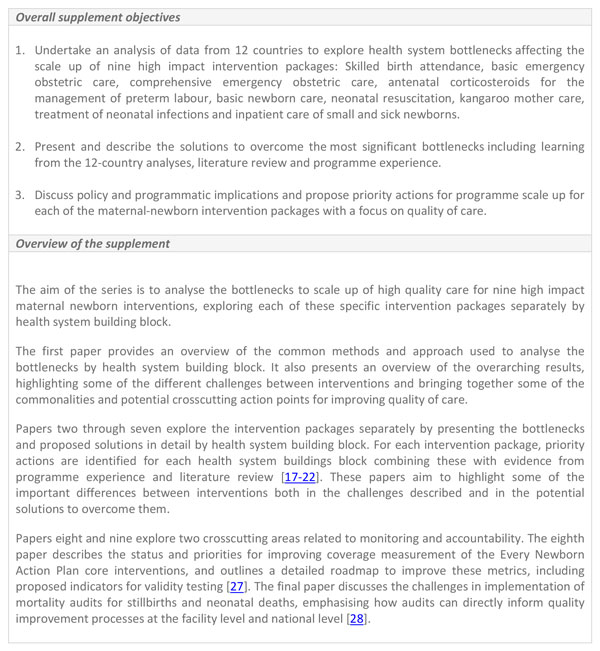
**Every Woman, Every Newborn: Supplement objectives and overview**.

Figure 1 outlines the objectives of the series overall and of the individual papers.

## Methods

We define a bottleneck as any factor that hinders or limits the ability of a health system to deliver the interventions as per recommended guidelines and therefore poses a barrier to delivering high quality maternal and newborn care to improve health outcomes.

### Country selection

**Figure 2:**
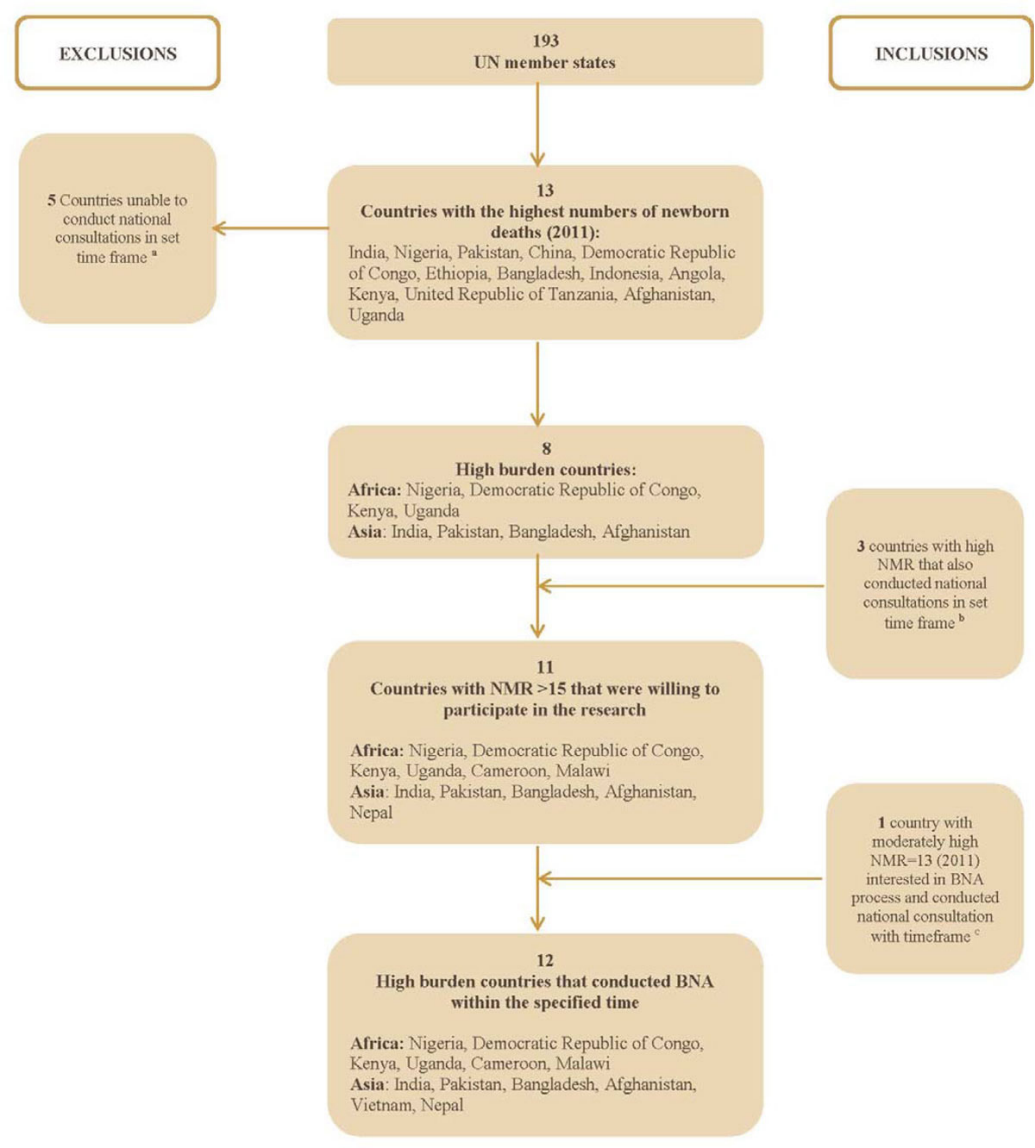
**Flowchart depicting country selection and analysis**. ^a^Angola, China, Ethiopia, Indonesia, and the United Republic of Tanzania ^b^Cameroon, Malawi, and Nepal ^c^Vietnam NMR: Neonatal mortality rate; BNA: Bottleneck analysis; UN: United Nations.

We included 12 countries in this systematic analysis, one third more countries than were included in the previous analysis of this data in *The Lancet *Every Newborn series [4]. The findings presented in this Series include data from six countries in Asia (Afghanistan, Bangladesh, India, Nepal, Vietnam and Pakistan) and six countries in Africa (Cameroon, Democratic Republic of Congo, Kenya, Malawi, Nigeria and Uganda) (Figure 2). The primary criteria for country selection was based on the number of births, number of newborn deaths, and neonatal mortality rate (NMR). We selected the top 13 countries with the highest numbers of newborn deaths in 2011. To ensure that we got a reasonable minimum set of data within a defined period and also to get a better understanding of the challenges that smaller high-burden countries might face, we also selected additional countries with high NMR (NMR ≥ 15). Vietnam was also included to increase the number of country perspectives from Asia. While Vietnam did not strictly fit into the criteria (Vietnam NMR was 13 in 2011), there was strong interest from the government of Vietnam to participate in the bottleneck analysis process.

### Data collection tool

The data were collected with the maternal-newborn bottleneck analysis tool which was compiled as part of the ENAP development process and is available online with instructions for completion (additional file 1) [24]. The tool aimed to facilitate the identification of bottlenecks that hinder the scale up of quality facility-based newborn care packages, as well as some maternal packages, across the six World Health Organization (WHO) health system building blocks [25] with community ownership and participation added on the basis of the recommendations of the Ouagadougou declaration on primary health care [26]. Nine maternal-newborn health facility-based high-impact intervention packages are identified in the tool [4]. For each package, specific tracer interventions were defined. These tracer interventions were chosen to represent the common challenges to implementing the package, to stimulate and focus discussion on identification of common challenges for components of the intervention delivered within the same time period (Table 1), the tracer interventions are described in detail in the intervention specific papers.

**Table 1:** Papers organised by intervention package showing differing priority health system building blocks.

Theme	Paper	Time of care		Tracer(s)	Health systems building blocks with most severe bottlenecks
Quality of care at birth for all newborns	2	*Labour and delivery*	Skilled Birth Attendance Basic Emergency Obstetric Care Comprehensive Emergency Obstetric Care	• Clean birth kits or delivery sets, oxytocin and partograph • Assisted vaginal delivery • Caesarean section and blood transfusions	Health workforce, health financing Health service delivery Health financing, health service delivery
	
	3	*Imminent labour*	Antenatal corticosteroids for management of mothers at risk of preterm labour	• Antenatal corticosteroids for fetal lung maturation	Health information systems, health service delivery, essential medical products and technologies
	
	4	*Immediate postnatal *	Essential Newborn Care Resuscitation	• Cleanliness, thermal control (including drying and wrapping, skin-to-skin contact, and delayed bathing) and support for breastfeeding • Bag and mask	Health financing, health service delivery Health workforce, essential medical products and technologies

Care of the small and sick newborns	5	*Postnatal *	Kangaroo Mother Care	• Not applicable	Leadership and governance, health financing, health workforce, health service delivery, community ownership and partnership
	
	6		Treatment of neonatal infections	• Injectable antibiotics	Health financing, health workforce, health information systems, community ownership and partnership
	
	7		Inpatient supportive care for sick and small newborns	• Intravenous fluids, feeding support, and safe oxygen	Health financing, health workforce, community ownership and partnership

Measurement and accountability	8	Indicators: Count every newborn: a measurement improvement roadmap for coverage data			
	
	9	Perinatal audit: Counting every stillbirth and neonatal death through mortality audit to improve quality of care for every pregnant woman and her baby			

### Data collection process

The bottleneck analysis tool was utilised during a series of national consultation workshops supported by the global *Every Newborn *Steering Group between July 1^st ^and December 31^st ^2013. The workshops were comprised of a group of national experts, mainly members of the maternal and newborn technical working group where existing, led by government and supported by a facilitating partner identified in each country [4]. In each workshop, after participants had identified the main bottlenecks to each tracer intervention for each health system building block, they came to consensus and graded the severity of the bottlenecks within each health system building block. The grading categories used were; good (not a bottleneck) (=1), needs some improvement (minor bottleneck) (=2), needs major improvement (significant bottleneck) (=3), or inadequate (**very major **bottleneck) (=4). Workshop participants also proposed potential strategies and solutions to overcome the priority bottlenecks identified under each health system building block. More details about the data collection process, workshops and participants are available in The Lancet *Every Newborn *Series and web appendix [4].

### Data analysis

For the purpose of this supplement, we present some of the intervention packages together as they are inextricably linked and care is provided for these packages across similar health systems platforms (Table 1). For example, skilled birth attendance (SBA), basic emergency obstetric care (BEmOC) and comprehensive emergency obstetric care (CEmOC) are presented together as the fundamental components of labour and birth. Basic newborn care (BNC) and neonatal resuscitation are usually provided by the same provider soon after birth. For the care of small and sick newborns, the bottlenecks to scale up of these intervention packages were extensive and distinct. Kangaroo mother care (KMC), treatment of neonatal infections and inpatient care of small and sick newborns are the cornerstones of care for small and sick newborns, but the factors hindering scale up varied across different countries. In order to uncover the nuances and different challenges for these interventions, they are presented in individual papers to allow for more in-depth analysis and discussion of the distinct challenges and potential solutions.

**Figure 3:**
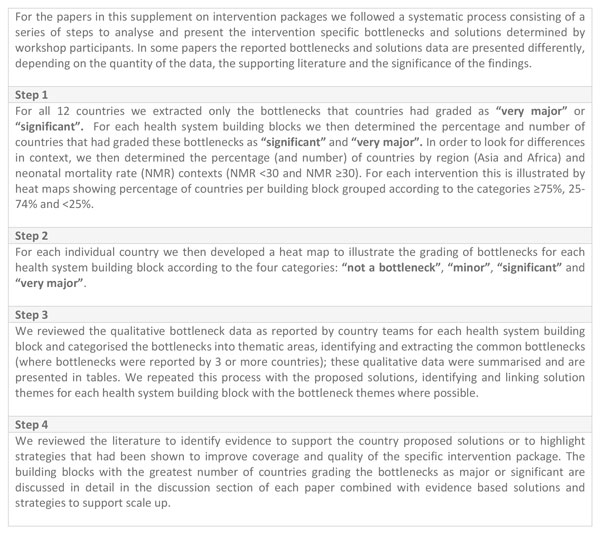
**Steps to analyse bottlenecks and solutions of maternal- newborn health interventions**. NMR: Neonatal mortality rate.

We followed a defined series of steps for each intervention packaged to identify and unpack the critical bottlenecks by health system building block and to compare bottlenecks between countries, regions and higher and lower mortality contexts, described in more detail (Figure 3). We surmised that the significant and major bottlenecks were the ones that were posing major barriers to scale up, therefore, out of over 3000 bottlenecks [4], across all the health system building blocks we only focused on the bottlenecks graded as significant or very major.

### Limitations

The bottleneck analysis tool was designed around specific high-impact intervention packages and tracers chosen to help elicit bottlenecks in a systematic way and assist comparison between countries. The tool had specific questions on the 'tracer' interventions (Table 1) to stimulate and focus discussions [4]. This might have constrained participants from thinking about bottlenecks more broadly and for other interventions within the package (beyond the identified tracers), some of which may have posed greater or different challenges to the scale up of the intervention package. The length of the tool (over 80 pages of questions) may have led to workshop fatigue resulting in some incomplete components in the questionnaires. In these cases, we worked with the in-country facilitating partners to assess the completeness of the data and, where possible, obtain additional information afterwards. We reviewed all the questionnaires submitted and informed the country facilitating partner when more information was needed. The facilitating partner worked with the government maternal newborn health (MNH) focal person and in some instances the Technical Working Group to review the workshop notes, provide further information where available and provide clarifications as requested.

The quality of the data from each country team was dependent on the skill of the facilitator to focus the discussion to reach consensus on bottlenecks, to apply appropriate grading for the bottlenecks within each building block and to encourage the group to discuss and propose innovative solutions. Some countries did not include grading for all of the building blocks, even where they listed bottlenecks and solutions, making the results more difficult to interpret. Similarly, for some countries, there are no solutions proposed. For example, Afghanistan did not provide any solutions for any of the intervention packages, which may have been due to a combination of workshop fatigue, facilitation issues and difficult conditions under which people worked at the time of analysis. The grading used for this analysis was generated from the consensus of those participating in the workshop. As the grades are based on views of the workshop participants and were not validated through an external process, they are subjective, but the workshop participants were drawn from broad areas of expertise within newborn health. However, there may have been some areas better represented than others and some countries with wider representation from different specialty areas; this might have affected the perception of the bottlenecks within each country and, subsequently, the findings in the analysis. Some workshop participants may have placed higher subjective value on certain health systems areas, or they may have viewed certain building block areas as easier challenges to overcome based on their knowledge of their setting or their specific area of expertise. However, given the consistency of our findings between countries we feel this was minimal. Due to time limitations, sometimes teams were split into different groups for the summary of the bottlenecks and solutions meaning that in some instances there is a misalignment between the bottlenecks described and the solutions offered. In our analysis, we have tried to link bottlenecks and solutions with the available evidence wherever possible.

The views expressed by the workshop participants do not necessarily represent that of the country as a whole. For this reason, wherever possible, we use the language "country teams" or "workshop participants" to present the interpretation of the results.

## Results

### Intervention-specific bottlenecks across the health systems building blocks

**Figure 4:**
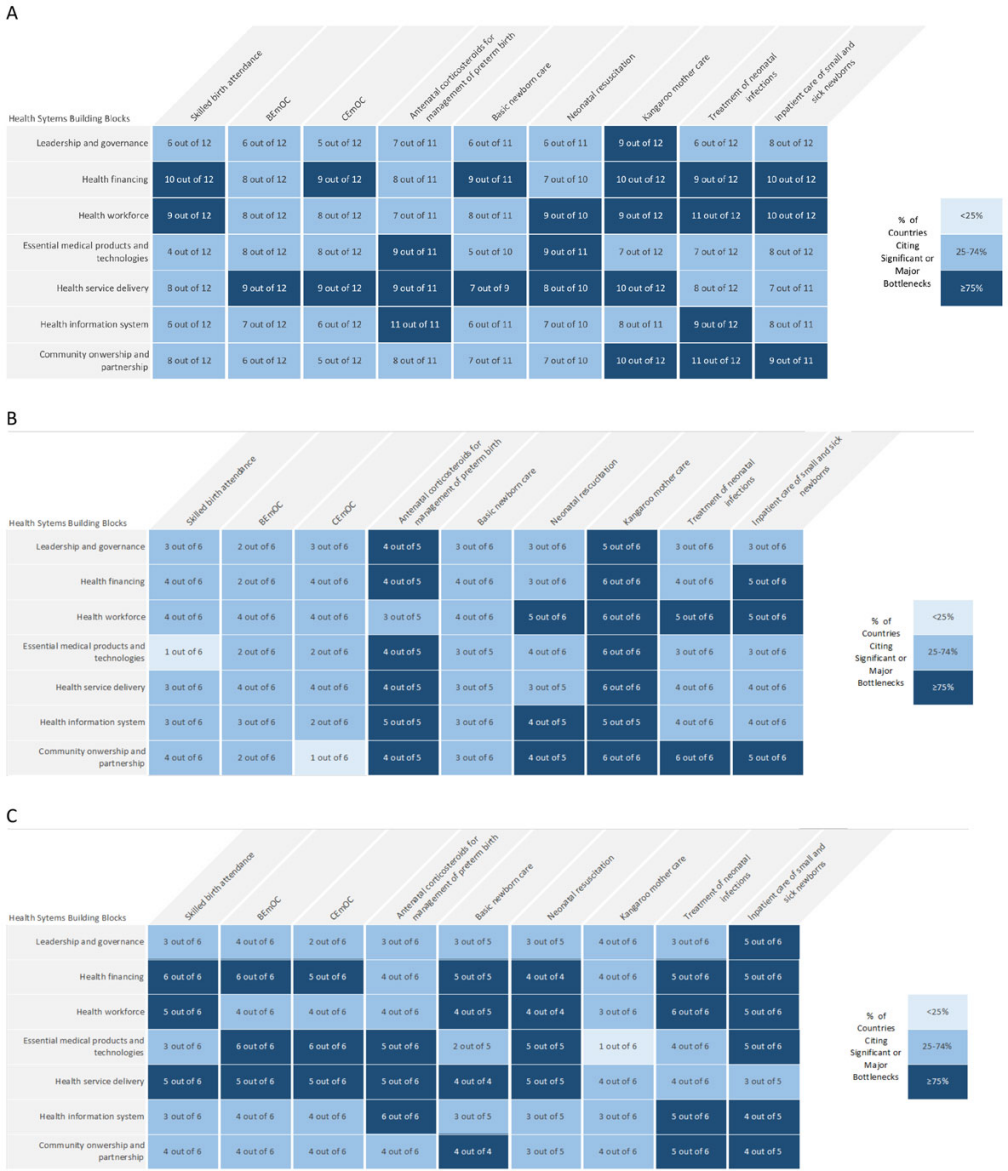
**Very major or significant health system bottlenecks for each maternal and newborn intervention**. Part A: All countries (n = 12)*: Afghanistan, Bangladesh, Cameroon, Democratic Republic of Congo, India, Kenya, Malawi, Nepal, Nigeria, Pakistan, Uganda, Vietnam. Part B: Asian countries (n = 6)*: Afghanistan, Bangladesh, India, Nepal, Vietnam and Pakistan. Part C: African countries (n = 6)*: Cameroon, Democratic Republic of Congo, Kenya, Malawi, Nigeria and Uganda. BEmOc: basic emergency obstetric care; CEmOC: comprehensive emergency obstetric care.

Table 2 provides an overview of which health system building blocks are ranked the most severely for each intervention package by all countries. The health system building blocks most commonly experiencing significant or very major bottlenecks across all nine intervention packages, were health financing, health workforce and health service delivery. Figure 4a shows the grading and number of countries for each intervention package for all twelve countries overall. The management of preterm births with antenatal corticosteroids (ACS), KMC, management of severe infections, and inpatient supportive care were identified as having the most severe (highest number of significant or major bottlenecks) across the 12 countries. While all intervention packages had areas of the health system with significant bottlenecks, BEmOC and basic newborn care overall had less severely graded bottlenecks. The regional differences are striking with over half of the countries in Asia reporting prevention and management of preterm birth and KMC as major bottlenecks; whereas in Africa the most severe bottlenecks were within basic newborn care and neonatal resuscitation (Figure 4b &4c). Table 2 highlights some of the overarching priority actions to overcome these bottlenecks by health system building block; broad based findings across all the papers are synthesised in this section and more details available in specific papers [17-22,27,28].

**Table 2:** Priority actions for country implementation of the Every Newborn Action Plan (ENAP) to improve quality of care by health system building block.

Health Systems Building Blocks	Priority actions	Interventions (n = 9) where >75% of countries identified health system building block as a priority	Milestone for 2020
Leadership and Governance	• Develop national newborn action plans or strategies that could be standalone plans or an integral part of reproductive, maternal, newborn, child and adolescent health or broader health sector plans. • Clearly define targets for maternal mortality ratio and neonatal mortality and stillbirth rates in national plans in line with the global *Every Newborn *Action Plan (ENAP) and Ending Preventable Maternal Mortality strategy.	1 (KMC)	National plans and targets for reducing newborn mortality rate, stillbirth rate and maternal mortality ratio.

Health Financing	• Allocate specific line items for newborn care in national and subnational health budgets. • Ensure financial health protection schemes cover the costs of care for newborns.	6 (SBA, CEmOC, BNC, KMC, Treatment of infections, Inpatient care of small and sick newborns)	Budget lines and insurance schemes outlining care for newborns especially the small and sick newborns included in national plans.

Health Workforce	• Develop and implement long term (5 and 10 year) costed human resource plans that outline country strategies for the training, distribution and retention of health workers particularly midwives, neonatal nurses and neonatologists.	5 (SBA, Neonatal Resuscitation, KMC, Treatment of neonatal infections, Inpatient care of small and sick newborns)	Train and retain the health workforce to provide quality care around the time of birth.

Essential Medical Products and Commodities	• Ensure that national essential drugs and commodity lists include the maternal newborn drugs and commodities identified by the United Nations Commission on Life Saving Commodities. • Strengthen procurement and supply systems to improve availability of supplies.	2 (ACS, Neonatal resuscitation)	Essential drugs for maternal newborn interventions included in national drugs lists and strengthen procurement and supply systems.

Health Service Delivery	• Establish global standards for quality care around the time of birth and implement through adaptation to country specific models to ensure sustainability.	6 (BEmOC, CEmOC, ACS, BNC, neonatal resuscitation, KMC)	Establish and implement quality standards of care.

Health Information Systems	• Include ENAP core indicators in country-led health management information systems. • Establish audit mechanisms in countries ensuring a minimum perinatal dataset is defined. • Strengthen civil and vital registration systems (CVRS) in countries to ensure that every newborn receives a birth certificate.	2 (ACS, Treatment of infections)	ENAP core metrics in country Health Management Information System and establish perinatal audit mechanisms.

Community ownership and partnership	• Transform social norms to improve care seeking for mothers and newborns, and reduce perceptions of fatalism that all small and sick newborns will die. • Engage with communities to demand quality care for every woman and every newborn as a basic human right.	3 (KMC, Treatment of infections, Inpatient care of small and sick newborns)	Transform social norms to demand quality care for every mother and newborn.

ACS: antenatal corticosteroids; BEmOC: basic emergency obstetric care; BNC: basic newborn care; CEmOC: comprehensive emergency obstetric care; CVRS: civil and vital registration system; ENAP: every newborn action plan; KMC: kangaroo mother care; SBA: skilled birth attendance.

**Reference: **Milestones from Every Newborn Action Plan: http://www.everynewborn.org/every-newborn-action-plan.

### Leadership and governance

Our analysis identified leadership and governance as a priority bottleneck, primarily for KMC (Figure 4a), where attention to policy and guidelines was viewed as fundamental to programmatic change and scale up. While it was not graded poorly relative to other building blocks for all other intervention packages, our qualitative analysis of the descriptions of bottlenecks found the lack of clear, evidence-based policies was also frequently identified as a bottleneck, especially for ACS, KMC and inpatient care of small and sick newborns. Even where written guidelines existed, country teams highlighted the need for regular updates and coordinated dissemination and implementation, especially to the lower levels of the health system. As a crosscutting issue, country teams identified the need for national champions and leaders, advocating for funding and implementation of quality maternal and newborn health services, research and working in collaboration with professional bodies and national academic institutions.

### Health financing

Health financing was graded significant or very major by most countries across all interventions and was therefore identified as a critical bottleneck. Our analysis identified that BEmOC, ACS and neonatal resuscitation were the only intervention packages where health financing was not perceived as having very major bottlenecks by at least 75% of countries (although at least two thirds of countries identified major bottlenecks for these interventions too) (Figure 4a). Overall, more participants from African countries graded health financing as a major bottleneck compared to participants from Asian countries (Figure 4b and 4c). Across all packages of interventions country teams referred to the disproportionately low funding for essential interventions, high out-of-pocket expenses for care-seeking and the low importance, hence lack of funding, for newborn health in national budgets. They also identified that lack of ring-fenced funding for care at birth including for care of small and sick babies and the lack of long-term, predictable financing limited planning and scale up efforts.

The health financing bottlenecks described were especially apparent in interventions related to mothers and newborns with complications requiring extra care. Funding the care of small and sick babies was seen as prohibitively expensive. Even where interventions were more affordable, such as KMC, failure to include the set-up costs in the plans due to poor budgeting was perceived as a barrier. Financing challenges were also identified for the most basic provision of care for all babies, even the basic supplies for warmth and feeding support.

### Health workforce

The health workforce building blocks were considered critical and were graded especially poorly for interventions that require specialised skills and training: skilled birth attendance, newborn resuscitation, treatment of neonatal infections and inpatient care of small and sick newborns. Key bottlenecks identified across all interventions packages included poor competency of staff, a lack of trained staff overall, especially midwives, specialist nurses (identifying the lack of a neonatal nursing cadre) and doctors. For most interventions, country teams identified specific areas of care where tasks could potentially be shifted to lower level professionals and where attention to specific policies on staffing could make more rational use of existing staff skills for both maternal and neonatal care, such as aspects of care for small and sick newborns. Country teams proposed the use of skills-based training approaches as a way to improve health worker competencies and performance. Country teams also suggested the need for supportive supervision and mentoring programmes to further enhance competencies and skills.

### Essential medical products and technologies

Essential medical products and technologies were identified as a priority area to tackle for ACS and neonatal resuscitation. However, whilst it was not graded as frequently as a major or significant bottleneck for other intervention areas, the qualitative section of the data country teams consistently reported shortages of equipment and drugs for all of the newborn interventions. For most of the interventions, country teams highlighted the weaknesses in supply and procurement systems resulting in continuing stock-outs and major inefficiencies (e.g., the introduction of parallel systems to procure drugs resulting in wasting of money or poorer quality supplies). Shortages of supplies were highlighted by country teams even for the most basic supplies for basic newborn care. To overcome some of these challenges, country teams proposed including some of the essential drugs in the national drugs and commodities lists such as ACS (e.g. dexamethasone) for fetal lung maturation and chlorhexidine for cord care. Country teams identified the need for improved capacity for logistics management with appropriate specifications for all the drugs and equipment needed for newborns on facility inventories at all the relevant levels. Better use of existing information technology to manage logistics could support needs-based forecasting of supplies and dissemination to all levels of the system.

### Health service delivery

In the context of the bottleneck tool, health service delivery relates to the ability of the health system to deliver the interventions with quality, as well as provide access to care. In our analysis, service delivery was graded most severely (by our definition of >75% of countries) for all interventions except SBA, management of severe infections and inpatient care of small and sick newborns; although even for these interventions, at least 60% of countries did identify quality of service delivery as a severe bottleneck. For BEmOC, the analysis identified a real deficit in the availability of assisted vaginal delivery. Other intervention bottlenecks were the lack of permissive policies to allow lower level staff to take on appropriate tasks in order to improve access to the services such as the use of ward assistants and/or nursing auxiliaries in the care of KMC babies (e.g. assisting with positioning and feeding) or use of community health workers at health posts to administer a first dose of antibiotics. Most country teams described problems with space allocation within health facilities to manage complications and provide the extra care needed for small and sick newborns. Specific quality bottlenecks were the lack of clinical audits (maternal and perinatal), the lack of use of supervision check lists and in-built quality assessments and quality improvement mechanisms at facility level for all interventions to ensure adherence to basic minimum standards, and also lack of daily checks to ensure basic equipment was functioning.

### Health information system

The health information system was identified as a priority intervention area for ACS and for treatment of neonatal infections. Country teams reported the lack of standardised indicators and, subsequently, lack of programmatic and coverage data for maternal and newborn interventions (not limited to ACS and sepsis) that was integrated into national systems to allow for monitoring and evaluation of programmes at a facility, district and national level. For almost all interventions, teams noted the limited capacity at district and facility level to analyse the data leading to limited utilisation of available data for decision making and action.

### Community ownership and partnership

Our analysis identified community ownership and partnership as a priority area for sick newborns including the treatment of neonatal infections, KMC and inpatient care for sick newborns. Whilst important deficits were described for most interventions, especially the lack of culturally appropriate and context-specific education and health information materials, the most notable bottlenecks were related to the lack of community involvement in the design and delivery of care. Country teams viewed this partnership as necessary to reduce fatalism, create demand for high quality care, increase care-seeking and improve adherence to treatment and care. The need to involve men and the wider family in care for ensuring safe childbirth care at facilities and for the care of small and sick newborns - whether as outpatients or as inpatients within a facility was highlighted. The involvement of communities was viewed as necessary to improve referral systems through the use of existing community resources for transportation and referral of mothers and newborns between facilities and to health posts when needed.

## Discussion

National achievement of the ENAP mortality targets and coverage goals will rely on tackling specific health system bottlenecks to the scale up of quality care. The findings presented in this supplement outline the most critical bottlenecks for nine high impact intervention packages for mothers and newborns at and around the time of birth. We examine the bottlenecks for each intervention in detail and expand on our previous analysis to include data from 12 high burden countries that account for approximately 58% of the global burden of neonatal deaths, 48% of maternal deaths and 57% of stillbirths [1-3]. By conducting a more systematic in-depth analysis for each intervention package, we highlight the intervention-specific challenges that are present and discuss these in detail in individual papers by health system building block. The results confirm that there is the need to broadly target bottlenecks within specific health system building blocks, such as health workforce, health financing and service delivery [4]. However, these papers illustrate the challenges in more depth and highlight variation by intervention package. For example, the implementation pathways used to scale up kangaroo mother care face specific challenges, and varying socio-cultural factors will require tailoring solutions to the context [21]. While health workforce bottlenecks are present across all interventions, the cadre of workers needed to overcome these challenges is different for each intervention. For example, inpatient care of small and sick newborn requires attention to nursing skills in existing facilities (neonatal nursing cadre) [22], whereas many of the labour and birth workforce bottlenecks are related to shortages of trained midwives (among other factors) [17,19].

Critics of the building block model for health systems research argue that the approach neglects the whole system perspective by separating out the health system into silos and giving all the blocks artificial equal importance [23]. This analysis attempted to address this limitation by giving participants the opportunity to grade the bottlenecks within each of the building blocks. The building block approach provides a common scientific language and structure for research [29], and we were able to use this logical structure to elicit the bottlenecks to scale up of quality care for each intervention package, and use this data to suggest priority actions within specific areas of the health system. The subsequent papers in the Supplement describe the nuance and details of the specific actions for priority building blocks within each of the intervention packages and, where appropriate, describe linkages and interactions between building blocks. In this paper, we outline some of the commonalities in the overarching results and highlight some of the crosscutting solutions (Table 2).

### Bottlenecks and priority actions to improve the quality of care for every mother and every newborn

#### Health financing

The lack of investment in health systems strengthening in countries is well known [30] and almost all country teams identified health financing as a priority building block (Figure 4a and Table 1). The provision of high quality maternal and newborn health services at facilities requires adequate financing for operations, staff, medicines, supplies, equipment and food. Various financing strategies have been employed to improve access to and utilisation of maternity services that have shown promising results [31-33]. India was the only country in our analysis that did not grade health financing as a major or significant bottleneck for all of the interventions; this may reflect how recent policy changes in India have been successful in prioritising maternal and newborn health in their national budgets through a comprehensive health systems approach [22].

**Figure 5:**
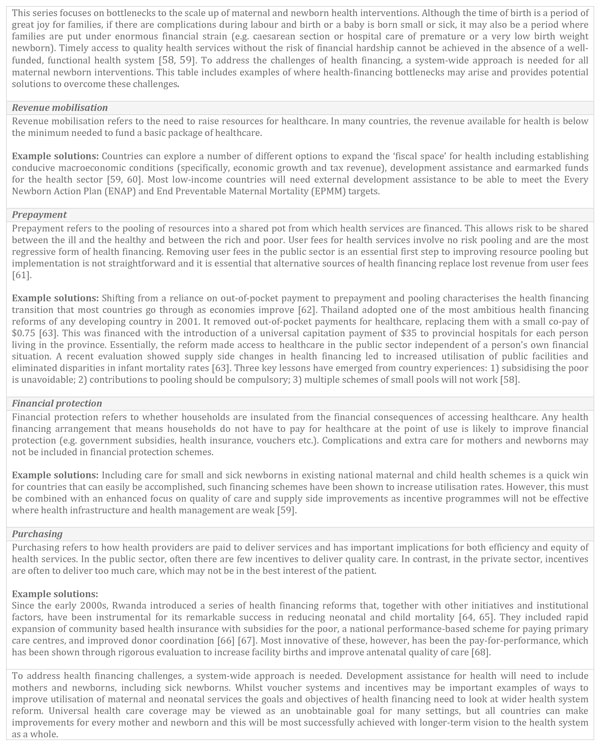
**Health financing as a health system bottleneck within the context of wider health sector reform**.

All countries in this analysis identified high out-of-pocket expenditure as a bottleneck, especially user fees. Country teams found that health financing affects the demand for care, especially for complicated pregnancies [17] and care of newborns that are small and sick [22]. Seeking care for these interventions at facilities has obvious implications for households in terms of transport costs, patient and their companions' time and their time away from work [17]. Figure 5 examines health financing as a bottleneck within the context of wider health system reform.

Many countries in Asia and Africa have pursued user fee removal or fee exemption for care during labour and birth, including for caesarean section [34]. However, appropriate financing strategies need to be extended for treatment of neonatal infections [20] and KMC [21], as well as comprehensive, inpatient special care, and ultimately neonatal intensive care [22]. To improve quality and access for the poorest and most vulnerable populations, national and local strategies to reduce out-of-pocket spending on health need to be developed [4], particularly ensuring that social health insurance schemes that provide free care for mothers and private health insurance for mothers also include care for newborns. This needs to be accompanied by increasing public awareness about the schemes and developing innovative enrolment strategies to reach out to the poorest and most vulnerable; specific strategies and examples for intervention packages are outlined in individual papers [17-22,28]. Context specific cost analysis and estimations of the financial burden placed on families when a baby is born small and sick are urgently needed to guide future policies and plans. Whilst teams referred to poor funding for care at and around the time of birth in national budgets overall, the budgeting, planning and rationalising of the cost of care for sick and small babies (especially moderate preterm) was especially needed, and viewed as a critical barrier.

The need for in-country guidance on the set-up costs and technical assistance on budgeting and planning processes for specific interventions for small and sick babies, such as KMC and special care units, was specifically highlighted.

#### Health workforce

Sufficient numbers of competent health care providers, including trained, licensed and regulated midwives and nurses, will be essential to deliver quality care resulting in the best outcomes [35]. There is growing consensus among public health professionals that midwifery care by educated, trained and licensed midwives has an essential contribution to make to high-quality maternal and newborn services and is associated with the more efficient use of resources, reduced mortality and improved quality of care for mothers and newborns [35-43]. Low and middle-income countries such as Burkina Faso, Cambodia, Indonesia, and Morocco have shown sustained and substantial reduction of maternal and newborn mortality while deploying midwives as a core constituent of their strategy [16]. However the ideal care for mothers and newborns, particularly when complications arise, requires a multi-disciplinary team including obstetricians, paediatricians, midwives, neonatal nurses and community health workers [44]. Nurses and midwives are at the front line of the response, and more need specialisation in neonatal care in order to respond to the demands on the health system, particularly for care of small and sick newborns.

To address the issue of distribution of staff, country teams identified a need for appropriate remuneration for staff and offered suggestions to improve daily working conditions, including provision of incentives for rural areas, covering food or transport costs and providing break-out areas for staff working long shifts. Better evidence is needed on workforce mobility e.g. how to measure and improve staff deployment, recruitment and retention, as well as posting and transfer of staff to remote and underserved areas [16]. WHO has provided guidelines to increase access and retain workers in rural and remote areas [45] and there have been attempts to outline new concepts for posting and transfer of staff [46].

For specific interventions packages, workshop participants gave examples of tasks that could be reorganised to make better use of human resources within their settings, but most of these have context-specific solutions based on existing health infrastructure, existing health workforce, culture and geography. For example, lower level health workers may be able to administer ACS to mothers at risk of imminent preterm birth in order to stimulate fetal lung maturation of babies <34 weeks gestation, but only in facilities with access to accurate gestational age assessment tools [18]. Health workers in the community require training to identify newborns with serious infections and initiate treatment before referral to higher level facilities [20]. For newborns that are preterm and may require prolonged stays in facilities, country teams suggested tasks (such as feeding and basic care) that could be shifted to nursing auxiliaries as well as to mothers, all of which require guidelines and inclusive policies that allow for involvement of mothers and family members [22], including KMC [21]. A recurrent theme across all interventions was the need for innovation to improve referral systems, using available resources, to ensure that mothers and newborns can be transferred to the appropriate level of care when needed [4,20,22].

Our findings suggest that countries need to develop long term (5 and 10 year plans) human resource plans that outline country strategies for the training, distribution and retention of health workers particularly midwives, neonatal nurses, obstetricians and neonatologists (Table 2). Specific skills are needed for those caring for small and sick newborns, and there is a lack of this specialised cadre in most settings [47]. Renfrew and colleagues [35] suggest that the planning for maternal and newborn care systems can benefit from using the quality framework in planning workforce development and resource allocation. The framework differentiates between what care is provided and how and by whom it is provided - attention needs to be paid to ensuring that all staff attending to women around the time of childbirth have the skills and competencies to care for the newborn as well. These plans and country policies need to also support investment in regulation, effective human resource management, and the service delivery environment in which health professionals work so that they will not only be able to cope with the increased workload, but will also ensure quality clinical and psychosocial care. Further work is needed to clearly determine how to improve the productivity and efficiency of the skilled workforce.

#### Health service delivery

Provision of accessible, quality services that are responsive to women's needs and wants should be part of the design of health-care service delivery [35]. The contact point of a skilled birth attendant is less effective without the full package of evidence-based, effective interventions around the time of birth including simple interventions, e.g. the monitoring of labour and the provision of basic newborn care, or more complex interventions, e.g. the provision of caesarean section and neonatal resuscitation.

**Figure 6:**
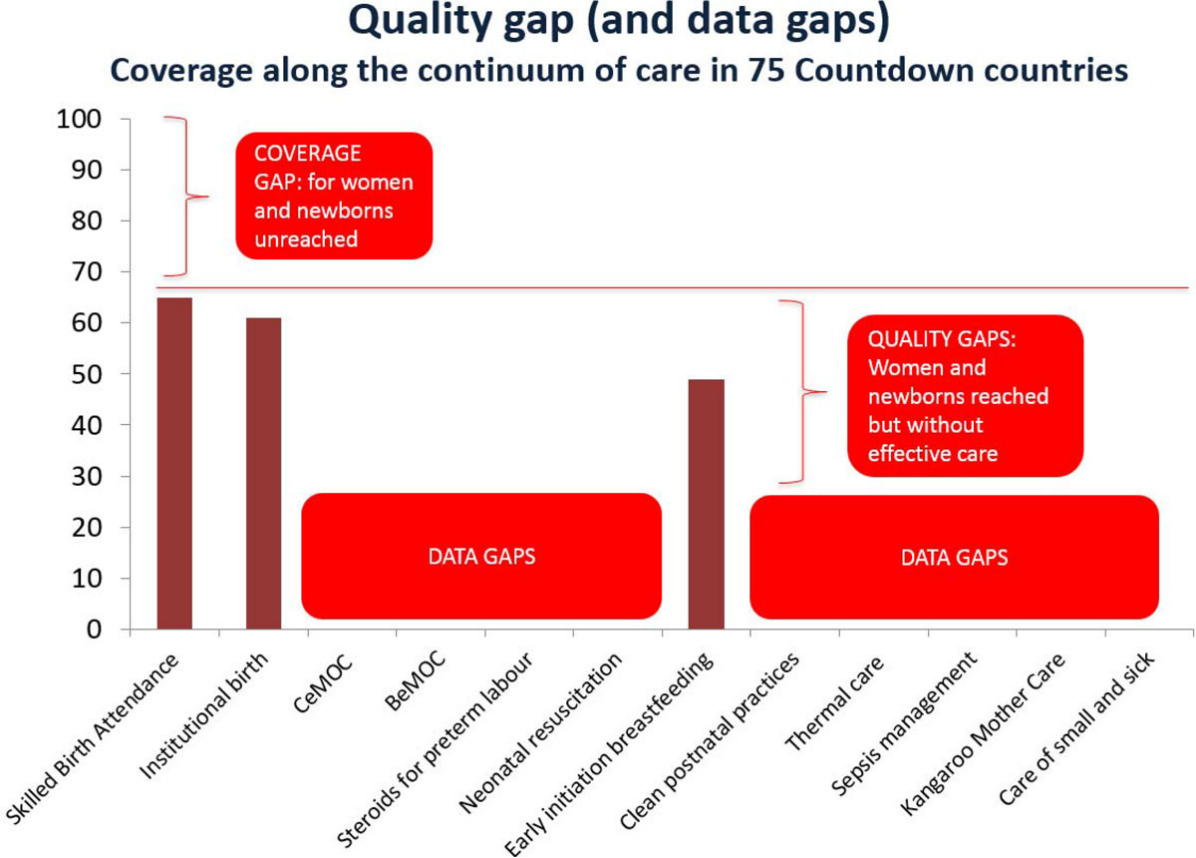
**Coverage of interventions around labour and childbirth and the quality and data gaps in 75 Countdown countries (median)**. Data sources: UNICEF. 2014. State of the World's Children 2015. Geneva: UNICEF. Adapted from Born Too Soon: Care for the preterm baby Joy E Lawn et al 2013 [69]. BEmOc: basic emergency obstetric care; CEmOC: comprehensive emergency obstetric care.

Figure 6 shows the coverage of skilled birth attendance in the 75 Countdown to 2015 priority countries (65%). The lack of data for most of these interventions flags the urgent need to improve metrics and include indicators in national health management information systems, as explored by Moxon et al in this supplement [27]. Weak systems for measurement of quality of care also affect the ability to identify and reduce such quality gaps. Maternal and perinatal mortality audits have proven to be useful to improve outcomes and quality of care, but only if the audit cycle is completed through to implementing solutions and evaluating outcomes [48]. All country teams proposed the scale up and effective use of audit data as a potential solution to improve the quality of care in facilities. The paper on mortality audits in this supplement presents examples of successful implementation highlighting the need for leadership for effective audit systems, and the development and use of clear guidelines and protocols in order to ensure that the audit cycle is completed [28].

### Quality care for every mother and every newborn

Scale up of quality care involves strengthening the dimensions this care; effectiveness, efficiency, access, safety, equity, appropriateness, acceptability and patient responsiveness or satisfaction in the care [15,49,50]. Both ENAP and EPMM prioritise the need to improve the quality of care for every mother and newborn. ENAP includes a specific milestone to develop a model for improving the quality of obstetric and newborn care in health facilities [6] and EPMM highlights a health systems and human-rights based approach towards quality of care emphasising availability, accessibility, acceptability and quality of services [8]. Taking forward the visions of ENAP and EPMM, the WHO presents a vision of a world where *"every pregnant woman and newborn receive quality care throughout pregnancy, childbirth and the postnatal period." *[50]. This is supported by a framework that identifies domains of quality of care which should be targeted to assess, monitor and improve care within the context of the health system as the foundation. Stillbirth rate is a uniquely specific, sensitive, measurable, and actionable indicator for the overall effective coverage of the continuum of quality of care - especially for antenatal and intrapartum care [2].

**Figure 7:**
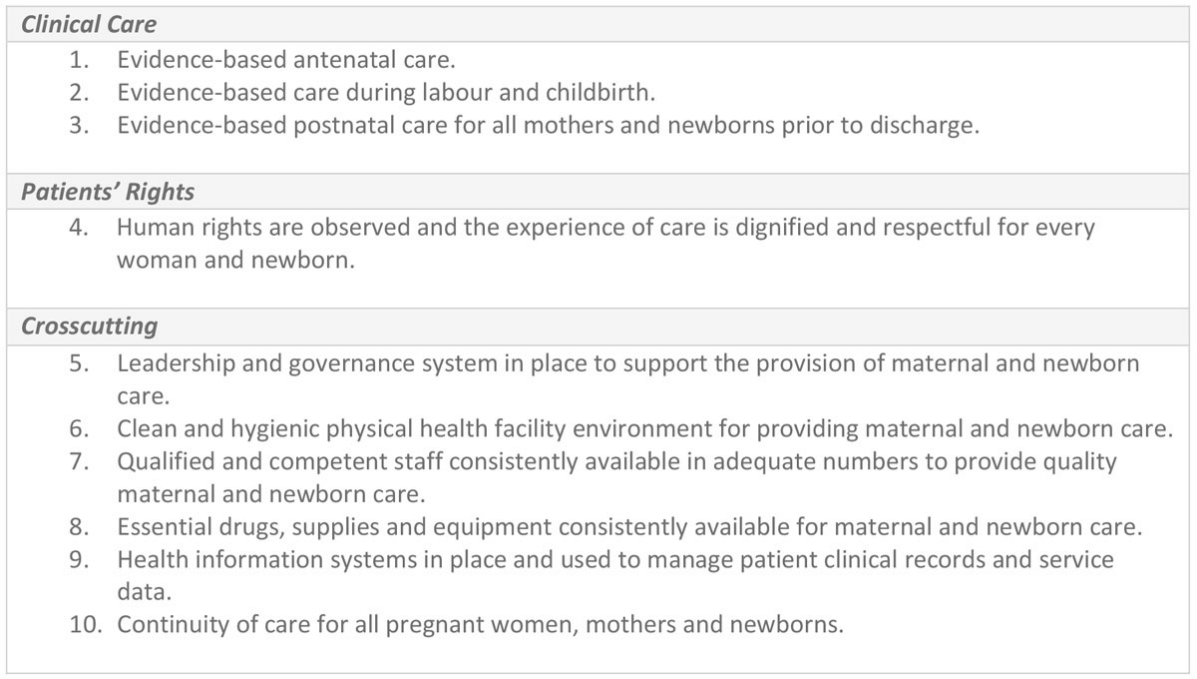
**Domain areas for improving quality care for mothers and newborns**.

The setting of quality standards will further support the improving and measuring of the quality of facility-based maternal and newborn care. Building on the WHO framework [50] and the health systems bottlenecks that need to be overcome to achieve quality care identified in this supplement, we propose 10 domain areas for maternal and newborn standards (Figure 7) related to the provision and experience of care outlined in the WHO framework. The clinical domain encompasses the high-impact interventions that will save most lives (Figure 7) [50]. A specific rights-based domain area is highlighted to emphasise the importance of the experience of the care in the WHO framework. Respectful maternity care is increasingly recognised as a critical element in quality health services. Evidence exists for the positive outcomes of having a companion of choice at the time of labour [51], emotional support [52], preferred birthing positions [53,54], a female provider [55], compassion by providers with adequate information exchange [56] and the encouragement of the parent's participation in care of their child in neonatal intensive care [57]. The areas proposed (Figure 7) also cover the relevant resources - human and financial - and supportive systems including the importance of leadership in quality improvement. These domain areas need to be translated into measurable standards and related criteria that can be incorporated into established country quality assurance mechanisms and sustainable systems.

## Conclusions

**Figure 8:**
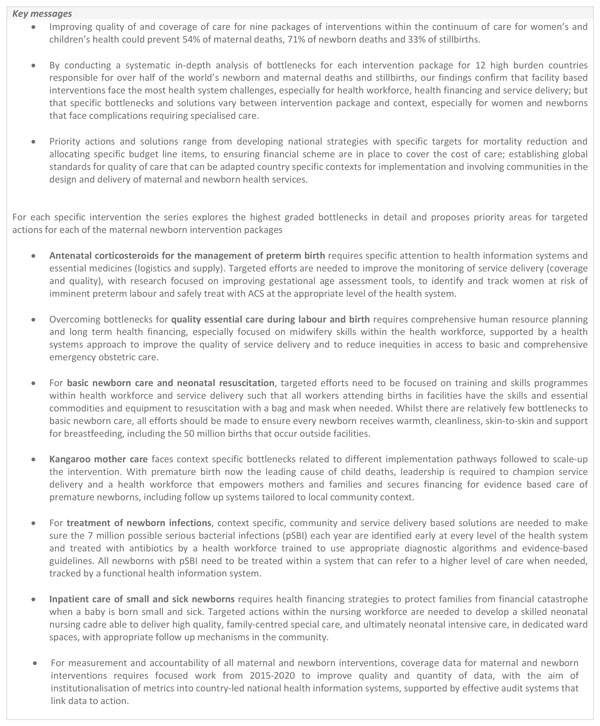
**Key messages**. ACS: Antenatal corticosteroids; pSBI: possible serious bacterial infection.

As the Millennium Development Goals era comes to a close, countries and global partners have recognised the need for greater attention to maternal, newborn, child and adolescent health as demonstrated by the development of action plans such as ENAP and EPMM. Ending preventable maternal and newborn deaths and stillbirths should firmly be part of the post-2015 development framework, including the Sustainable Development Goals (SDGs) as targets and indicators within the single health goal. These targets will not be achieved without improving the quality of care around the time of birth and for small and sick newborns. However, the gaps in the quality of essential maternal and newborn care remain a major challenge, and unless urgently addressed, nearly 2 million lives of women and their babies will be lost each year [5]. Key messages from the series are summarised in Figure 8. The survival of newborns (especially those who are small and sick), who can die in minutes, depends on the health system response and their survival can be considered as a sensitive test of the quality of care the health system provides.

Moving forward to the post-2015 agenda, a concerted, systematic and targeted approach is needed to strengthen health systems with a focus on the context and intervention-specific bottlenecks preventing the scale up of those interventions that have the potential to save the most lives. The broad strategies and solutions proposed in this paper, and the intervention specific solutions outlined in papers throughout the supplement provide guidance to countries to facilitate action to prevent maternal and newborn deaths and stillbirths.

## List of abbreviations

ACS: Antenatal Corticosteroids; BEmOC: Basic Emergency Obstetric Care; BNC: Basic Newborn Care; CEmOC: Comprehensive Emergency Obstetric Care; EmOC: Emergency Obstetric Care; ENAP: Every Newborn Action Plan; EPMM: Ending Preventable Maternal Mortality; KMC: Kangaroo Mother Care; MNH: Maternal and newborn health; NMR: Neonatal Mortality Rate; pSBI: possible serious bacterial infection; RMNCAH: Reproductive, maternal, newborn, child and adolescent health; SBA: Skilled Birth Attendant; WHO: World Health Organisation.

## Competing interests

All authors declare they have no competing interests. The assessment of bottlenecks expressed during consultations reflects the perception of the technical experts and may not be national policy. The authors alone are responsible for the views expressed in this article and they do not necessarily represent the decisions, policy or views of the organisations listed, including WHO.

## Authors' contributions

KED, JEL, MVK and SGM conceptualised the paper and coordinated the writing process. KED and AS-K coordinated the tool development and country consultation process. MVK, SGM, AS-K, CN and KED were responsible for the analysis and figures. All named authors contributed to paper drafts and approved the final manuscript.

## Supplementary Material

Additional file 1 Click here for file

## Declarations

Publication costs for this supplement was funded by the Bill and Melinda Gates Foundation through a grant to the US Fund for UNICEF (Grant ID: OPP1094117), and support from Save the Children's Saving Newborn Lives Programme. Additional funding for the country consultations was received from USAID (Grant ID: GHA-G-00-07-00007) through UNICEF.

This article has been published as part of *BMC Pregnancy and Childbirth *Volume 15 Supplement 2, 2015: Every Woman, Every Newborn. The full contents of the supplement are available online at http://www.biomedcentral.com/bmcpregnancychildbirth/supplements/15/S2.
