# Supplementary material for: Scaling up quality care for mothers and newborns around the time of birth: an overview of methods and analyses of intervention-specific bottlenecks and solutions
Source: BMC Pregnancy Childbirth. 2015 Sep 11;15(Suppl 2):S1. doi: 10.1186/1471-2393-15-S2-S1 (PMC4578819; doi:10.1186/1471-2393-15-S2-S1)
Supplement: Additional file 1 [file 1471-2393-15-S2-S1-S1.docx]

Scaling up quality care for mothers and newborns around the time of birth: an overview of methods and analyses of intervention-specific bottlenecks and solutions

Additional file 1

A. Bottleneck tool questionnaire 2

# Bottleneck tool questionnaire

**TOOL TO SUPPORT COUNTRIES TO IDENTIFY**

**BOTTLENECKS AND SOLUTIONS TO SCALE-UP NEWBORN CARE**

This tool is divided into 2 main sections:

**SECTION I: IDENTIFICATION OF BOTTLENECKS APPLICABLE TO ALL NEWBORN INTERVENTIONS**

Section I includes questions related to newborn health programmes in general and is organized into 7 sub-sections according to the health system building blocks as follows:

1. Leadership and governance
2. Health finance
3. Health workforce
4. Health service delivery
5. Essential medical products and technologies
6. Health information systems
7. Community ownership and partnership

At the end of each health system building block, summarize the key bottlenecks and provide an assessment of whether the health system area is:

- **Good** *(not a bottleneck to scale up)*
- **Needs some improvements** *(minor bottleneck to scale up)*
- **Needs major improvements** *(significant bottleneck to scale up)*
- **Inadequate** *(very major bottleneck to scale up)*

**SECTION II: IDENTIFICATION OF BOTTLENECKS APPLICABLE TO EACH CRITICAL NEWBORN INTERVENTION**

Section II is sub-divided into 9 sections representing critical newborn interventions listed below. Bottlenecks specific to each essential intervention are also assessed according to the 7 health system building blocks.

1. Management of pre-term birth focus on antenatal corticosteroids
2. Skilled care at birth focus on the use of the partograph
3. Basic Emergency Obstetric Care focus on assisted vaginal delivery
4. Comprehensive Emergency Obstetric Care focus on caesarean section
5. Basic Newborn Care focus on cleanliness/cord care, warmth, and feeding
6. Neonatal resuscitation
7. Kangaroo mother care focus on skin to skin, breastfeeding and feeding support

for premature and small babies

1. Treatment of severe infections focus on using injectable antibiotics
2. Inpatient supportive care for focus on IV fluids/feeding support and safe oxygen

for sick and small newborns

At the end of each health system building block, summarize the key bottlenecks and provide an assessment of whether the health system area is:

- **Good** *(not a bottleneck to scale up)*
- **Needs some improvements** *(minor bottleneck to scale up)*
- **Needs major improvements** *(significant bottleneck to scale up)*
- **Inadequate** *(very major bottleneck to scale up)*

**EACH SECTION INCLUDES A CHAPTER ON STRATEGIES AND SOLUTIONS TO ADDRESS IDENTIFIED BOTTLENECKS**

Potential and successful strategies and solutions will be identified by priority bottlenecks under each health system building block.

***How to conduct an analysis of bottlenecks and solutions to scale-up newborn care***

***Data collection process including data sources***

The country Maternal and Newborn Health (MNH) Technical Working Group (TWG) should coordinate the collation of documents relevant to the 9 interventions prior to the workshop. A focal point/consultant should be identified by the TWG to support the data collation.

Relevant documents and data sources to collate include national RMNCH strategies/plans/policies, national guidelines and standards related to the 9 interventions, periodic reports, reviews, RMNCH needs assessments, and existing country survey data. For example, countries that have already done the Rapid Landscape Analysis of RMNCH Interventions and Commodities (RAIC) will have a substantial amount of information that can be used to provide background information and prefill the tool. Additional data will be available from the DHS/MICS, SARA, Countdown, EmONC assessments, and other available MNH surveys.

**Participatory data review and analysis**

- Participants should be divided into Working Groups to complete the tool. Ensure a good mix of working group participants in each group including maternal and newborn program planners, district level personnel and members of civil society.
- Try to keep the groups to a maximum of 8-10 participants. Interventions that can be grouped together include: *Section I , Interventions 2, 3,and 4 – focus on maternal health; Interventions 5 and 6 – focus on immediate and essential newborn care; Interventions 1 and 7 – focus on prematurity; Interventions 8 and 9 – focus on the sick newborn.*
- Participants from each intervention work group will examine each of the 7 health system building blocks to elicit the key bottlenecks to scale up of the intervention per building block. At the end they will need to summarize the key bottlenecks for each health system building block per intervention.
- Participants will then determine the relative impact of the health system bottlenecks on the scale up of newborn programmes in general and on critical newborn interventions in particular. The grading will be a subjective assessment made by a consensus according to the scale provided - good to inadequate.
- After identifying the bottlenecks in Section I and each intervention in Section II, each group should prioritize the intervention bottlenecks they have identified for each health system building block and identify potential solutions to overcome them. The solutions should be feasible (with clear milestones), cost-effective, equity-focused and sustainable.

***Validation of results and submission of findings to the core group***

- Each group will feedback the bottlenecks and solutions during a plenary session to all participants for validation. Presentations will be collated and synthesized into one short country report summarizing key bottlenecks to scale-up of newborn care as well as evidence-based solutions and actions to address the challenges. This could be done by the TWG/MNH consultant in close collaboration with the member of the core group who provided technical assistance to the country team.
- All the raw data should also be provided to the global *Every Newborn* Core Group.

| ***SECTION I: QUESTIONS TO IDENTIFY HEALTH SYSTEM BOTTLENECKS***  ***APPLICABLE TO ALL NEWBORN INTERVENTIONS*** |
| --- |

**For each of these questions described below, please conduct an in-depth analysis of your answer and provide detailed explanations (reasons) to back up your response.**

| **1. LEADERSHIP AND GOVERNANCE** | |
| --- | --- |
| 1.1 Does the national RMNCH strategy identify averting neonatal deaths and improving newborn health, in general, as a priority?   - Is there a situation analysis of newborn health? If not, why? - Is there a baseline figure for the neonatal mortality rate? - Is there a specific target for NMR and/or early NMR? By which date? | |
| 1.2 Explain how the national RMNCH strategy identifies and addresses the leading causes of neonatal mortality (prematurity, asphyxia, infection) as priority RMNCH interventions. | |
| 1.3 Who is the MoH focal person for newborn health? If a MoH focal point for newborn health does not exist, please explain why not.  Specify the department(s) in which the person is located. Even if there is one focal person please assess whether this is sufficient to cover the national needs. Summarize the current challenges.  How about at district level, are there focal persons? | |
| 1.4 Describe the functional national coordination mechanism/ technical working group/ national steering committee addressing newborn health. Please list key stakeholders and describe the regularity of meetings, specifically meetings on reviews of progress on newborn health. If such a mechanism/group does not exist, please explain why. | |
| 1.5 Describe the country’s birth registration policy, whether it is mandatory and if the birth certificate is free of charge. | |
| 1.6 Please explain if District Health Management Teams are able to take decisions on planning and management of resources for newborn health. | |
| **Please provide a summary of key bottlenecks.** |  |
| **After responding to the questions above, please make an overall assessment of whether leadership and governance for newborn programmes is:**   - **Good** *(not a bottleneck to scale up)* - **Needs some improvements** *(minor bottleneck to scale up)* - **Needs major improvements** *(significant bottleneck to scale up)* - **Inadequate** *(very major bottleneck to scale up)* |  |
| **2. HEALTH FINANCING** | |
| 2.1 Describe the current national funding mechanisms for newborn health (programmes and commodities). What proportion comes from external/donor resources? | |
| 2.2 Describe if the budget allocated for maternal and newborn health services in 2011-2012 was sufficient. If not, please explain why. | |
| 2.3 Describe the policy for free care at point of delivery for women and their newborns. Describe other national programmes (e.g. national funding scheme, voucher programmes, etc.) to facilitate free care. Please specify the funding source (government, donors). If such a policy does not exist, please explain why. | |
| 2.4 What extra fees (unofficial) do women have to pay in addition to the official fees? Please explain. | |
| 2.5 Describe the results-based financing mechanism in place to rapidly increase access to maternal and newborn services to the most in need (poorest). What is covered? How widespread it is? Please mention the regions or districts where this is in place. | |
| **Please provide a summary of key bottlenecks.** |  |
| **After responding to the questions above, please make an overall assessment of whether health financing mechanisms for newborn programmes are:**   - **Good** *(not a bottleneck to scale up)* - **Need some improvements** *(minor bottleneck to scale up)* - **Need major improvements** *(significant bottleneck to scale up)* - **Inadequate** *(very major bottleneck to scale up)* |  |
| **3. HEALTH WORK FORCE** |  |
| 3.1 Describe the national human resource policy that addresses the needs of MNH for the cadres and situations listed below. Please specify the name and date/period of the document.     - Midwifery personnel for care at birth: - Community health workers (CHW) for home-based maternal and newborn care: - Appropriate skill mix of personnel for facility-based care for sick newborns: |  |
| 3.2 Explain how the national human resource policy addresses the following strategies for scaling up childbirth and newborn care. If it does not, please explain why for each strategy.   - Improving HR capacity (training and deployment, skill mix) for:   Midwifery personnel? *Please explain*  Nursing small and sick neonates? *Please explain*   - Financial mechanisms for:   Motivation and retention? *Please explain*  Incentivizing work in remote areas? *Please explain* |  |
| 3.3 Please, specify the following:  *Is the current staffing situation sufficient? What are the percentages of unfilled posts for each category?*  **Staff Complement % Unfilled posts**  Overall health worker density (number/10,000 population)  Physician density (number/10,000 population)  Nurse density (number/10,000 population)  Midwife density (number/10,000 population)  Community worker density (number/10,000 population) |  |
| **Please provide a summary of key bottlenecks.** |  |
| **After responding to the questions above, please make an overall assessment of whether human resources for newborn programmes are:**   - **Good** *(not a bottleneck to scale up)* - **Need some improvements** *(minor bottleneck to scale up)* - **Need major improvements** *(significant bottleneck to scale up)* - **Inadequate** *(very major bottleneck to scale up)* |  |
| **4. ESSENTIAL MEDICAL PRODUCTS AND TECHNOLOGIES** | |
| 4.1 Describe the national coordination mechanism for procurement and supply chain (PSM) management. If one does not exist, please explain why. | |
| 4.2 Explain whether the logistics management system includes essential commodities for newborns. Briefly describe the type (e.g. manual, enterprise software) and the furthest level the system can track (national, regional, district, health facilities). If it does not include essential commodities for newborns, please explain why. | |
| **Please provide a summary of key bottlenecks.** |  |
| **After responding to the questions above, please make an overall assessment of whether PSM of commodities for newborn programmes is:**   - **Good** *(not a bottleneck to scale up)* - **Needs some improvements** *(minor bottleneck to scale up)* - **Needs major improvements** *(significant bottleneck to scale up)* - **Inadequate** *(very major bottleneck to scale up)* |  |
| **5. HEALTH SERVICE DELIVERY** | |
| - 1. Describe the country’s national policy on quality improvement for maternal and newborn health services. Please specify the name and date of the document. | |
| 5.2 Describe the systems in place for reviewing competencies and re-certification of the following key personnel providing maternal and newborn care. If none exists, please explain why.  Midwifery personnel? *Please explain*  Nursing small and sick neonates? *Please explain* | |
| 5.3Describe the country’s system in place for routine supervision of (1) hospitals and (2) health facilities at the following levels. If a system does not exist, please explain why.  (a) District level  (b) National level | |
| **Please provide a summary of key bottlenecks.** |  |
| **After responding to the questions above, please make an overall assessment of whether health service delivery for newborn programmes is:**   - **Good** *(not a bottleneck to scale up)* - **Needs some improvements** *(minor bottleneck to scale up)* - **Needs major improvements** *(significant bottleneck to scale up)* - **Inadequate** *(very major bottleneck to scale up)* |  |
| **6. HEALTH INFORMATION SYSTEMS** | |
| 6.1 According to the national legal requirements, are all foetuses and infants weighing at least 500 g at birth (or 22 completed weeks or 25 cm crown-heel length), whether alive or dead, included in the national statistics? ☐ yes ☐ no If no, please indicate which different criteria are being applied | |
| 6.2 Describe the country’s functional national health management information system (HMIS) (timeliness, completeness, accuracy, etc.). If one does not exist, please explain why. | |
| 6.3 Describe the newborn health related data collected by HMIS. Please indicate if the following data is included:   - Facility-based (early) neonatal mortality - Disaggregated by birth weight categories - Neonates protected at birth against neonatal tetanus - Proportion of newborns who started breastfeeding within 1 hour - Proportion of newborns receiving hepatitis B vaccination birth dose within 24 hours of birth - Proportion of newborns (0-1 month) exclusively breastfed - Others- please specify the indicators.   For each indicator, please specify how and how frequently they are collected. | |
| 6.4 Describe and provide details of the country’s functional national system of accountability for reporting of progress in MNH and the oversight in place (e.g. annual score card). If such a system does not exist, please explain why. | |
| - 1. Describe the country’s functional Death Surveillance Response mechanism Specify if it covers maternal and perinatal deaths (including stillbirths and neonatal deaths). If this does not exist, please explain why. | |
| 6.6 Describe the validation mechanism system in place to ensure high quality data reporting. | |
| 6.7 Explain if newborn data are available from private health care facilities and at what level the information is compiled. Please explain the mechanism. | |
| **Please provide a summary of key bottlenecks.** |  |
| **After responding to the questions above, please make an overall assessment of whether the health information system for newborn programmes is:**   - **Good** *(not a bottleneck to scale up)* - **Needs some improvements** *(minor bottleneck to scale up)* - **Needs major improvements** *(significant bottleneck to scale up)* - **Inadequate** *(very major bottleneck to scale up)* |  |
| **7. COMMUNITY OWNERSHIP AND PARTNERSHIP** |  |
| 7.1 Describe the demand generation/behaviour change communication initiatives included in the national RMNCH plan. Or describe the country’s national communication and behaviour change strategy focusing on newborn health. If none exist, please explain why. |  |
| 7.2 Describe existing functional community engagement mechanisms/initiatives (e.g. women groups, community representatives in health facility management committees, community members in accreditation committees, etc.). Are these nationwide or just in pilot sites? |  |
| **Please provide a summary of key bottlenecks.** |  |
| **After responding to the questions above, please make an overall assessment of whether community ownership and participation for newborn programmes is:**   - **Good** *(not a bottleneck to scale up)* - **Needs some improvements** *(minor bottleneck to scale up)* - **Needs major improvements** *(significant bottleneck to scale up)* - **Inadequate** *(very major bottleneck to scale up)* |  |

**IDENTIFICATION OF SOLUTIONS** **TO ADDRESS THE CHALLENGES**

*Please add sheets as appropriate*

| ***NEWBORN CARE IN GENERAL*** | | |
| --- | --- | --- |
| **Summary of key bottlenecks *by order of priority*** | | Strategies and solutions to address identified challenges and bottlenecks |
| *Building block* | *Priority bottlenecks* |  |
| *Leadership and Governance* |  |  |
| *Health Finance* |  |  |
| *Health Workforce* |  |  |
| *Essential Medical products and Technologies* |  |  |
| *Health Service Delivery* |  |  |
| *Health Information Systems* |  |  |
| *Community Ownership and Participation* |  |  |

| ***SECTION II: QUESTIONS TO IDENTIFY HEALTH SYSTEM BOTTLENECKS***  ***APPLICABLE TO EACH CRITICAL NEWBORN INTERVENTION*** |
| --- |

**Intervention 1: *PREVENTION AND* *MANAGEMENT OF PRETERM BIRTH***

***Focus on the use of antenatal corticosteroids***

The prevention and management of preterm birth includes a number of interventions such as the detection of the woman at risk or already in preterm labour, the use of tocolytics or a different route of delivery. However, the use of antenatal corticosteroids for foetal lung maturation has been identified as the tracer intervention, for the purpose of this exercise, as it has the highest potential impact on mortality and morbidity.

| **1. Leadership and governance** |
| --- |
| 1.1 Specify the name of the document(s) as part of the national RMNCH plan/strategy where prematurity has been identified as a major cause of preventable deaths. Explain if the plan/strategy includes specific actions to avert those deaths through prevention and management of preterm birth, including the use of antenatal corticosteroids. |
| 1.2 Describe the national standard treatment guidelines or clinical protocols covering prevention and management of preterm birth Please specify name of the guidelines/clinical protocol document and year of publication. If none exist, please explain why.   - Do they contain a recommendation on the use of antenatal corticosteroids for foetal lung maturation? - Are they in line with current best practices (e.g. latest WHO guidelines)? - Are they regularly updated? Specify the dates of the last revision. |
| 1.3 If the use of antenatal corticosteroids for foetal lung maturation is recommended, please specify the level of care. Please relate your country health facility levels of care to the ones below:   - Primary level - Primary level only as pre-referral dose - First referral (secondary) level - Second referral (tertiary level), e.g. only where specialized OBGYN care is available |
| 1.4 If the use of antenatal corticosteroids for foetal lung maturation is recommended, explain if relevant policies or regulations are aligned with these recommendations. For example:   - Explain if all health workers who manage women in preterm labour are authorized to prescribe and administer antenatal corticosteroids. (more details in section on human resources) - Explain if the policies on the procurement and distribution of medicines are aligned with the recommendation.(more details in the section on essential medical products and technologies) |
| **Please provide a summary of key bottlenecks.** |
| **After responding to the questions above, please make an overall assessment of whether leadership and governance for the prevention and management of preterm birth, including the use of antenatal corticosteroids antenatal corticosteroids is:**   - **Good** *(not a bottleneck to scale up)* - **Needs some improvements** *(minor bottleneck to scale up)* - **Needs major improvements** *(significant bottleneck to scale up)* - **Inadequate** *(very major bottleneck to scale up)* |
| **2. Health Financing** |
| 2.1 Describe specific health care financing issues related to the procurement and/or distribution of antenatal corticosteroids for the intended level of care, for example:   - *At the national level:*   - Describe if choice of the recommended drug (dexamethasone vs. betamethasone, *please specify which drug*) is adequate for the level of funding.   - In centralized procurement and distribution systems, describe if the available funds are sufficient to procure and distribute, continuously and sustainably, antenatal corticosteroids to all health facilities where they are required. - *At the district or facility level:*   - Describe if the choice of the recommended drug (dexamethasone vs. betamethasone) poses a problem.   - In decentralized procurement and distribution systems, explain if the available funds are sufficient to procure, continuously and sustainably, antenatal corticosteroids at facility or district level. |
| 2.2 Describe any financial barriers for the women who should receive antenatal corticosteroids, to benefit from this intervention. For example:   - Does this medicine need to be paid for in addition to the general consultation or treatment fees? - Do patients/families need to purchase antenatal corticosteroids themselves because they are not provided by the health care provider/facility? - Does the price of the recommended drug present a financial barrier? - Are there any additional fees associated with this intervention (e.g. the need to pay for a prescription or a specialist consultation fee)? |
| 2.3 Describe other financial barriers to the scale up this specific intervention. |
| **Please provide a summary of key bottlenecks.** |
| **After responding to the questions above, please make an overall assessment of whether health financing for the prevention and management of preterm birth, including the use of antenatal corticosteroids, is:**   - **Good** *(not a bottleneck to scale up)* - **Needs some improvements** *(minor bottleneck to scale up)* - **Needs major improvements** *(significant bottleneck to scale up)* - **Inadequate** *(very major bottleneck to scale up)* |
| **3. Health workforce** |
| 3.1 Explain whether there are sufficient numbers of competent health care workers who can give antenatal corticosteroids, at each level of care, where this intervention should be implemented. Issues may include:   - There are not sufficient midwifery personnel who can provide skilled care at birth, including recognizing preterm labour. - There might be sufficient numbers of skilled and motivated health care workers, but they are not authorized to prescribe or administer antenatal corticosteroids. - There might be sufficient numbers of motivated health care workers, but they do not have the necessary competencies for the appropriate use of antenatal corticosteroids. |
| 3. 2 What cadre of health care workers are authorized to prescribe and/or administer antenatal corticosteroids?     - Nurses ☐Administer only ☐Prescribe and administer ☐ Prescribe only - Midwives ☐Administer only ☐Prescribe and administer ☐ Prescribe only - Associate clinicians: ☐Administer only ☐Prescribe and administer ☐ Prescribe only - Non-specialist doctors: ☐Administer only ☐Prescribe and administer ☐ Prescribe only - Other cadre: …………. ☐Administer only ☐Prescribe and administer ☐ Prescribe only   *Please specify (e.g. auxiliary midwives/nurses)* |
| 3. 3 Explain whether there are (standard) job descriptions for health workers for all levels of care that reflect their role in prescribing and/or administering antenatal corticosteroids. If none exists, please explain why. |
| 3.4 Are there competency-based training programmes through which the respective cadre of health care workers listed below acquire the necessary knowledge and skills to prescribe and or administer antenatal corticosteroids?   - Nurses ☐Administer only ☐Prescribe and administer ☐ Prescribe only - Midwives ☐Administer only ☐Prescribe and administer ☐ Prescribe only - Associate clinicians: ☐Administer only ☐Prescribe and administer ☐ Prescribe only - Non-specialist doctors: ☐Administer only ☐Prescribe and administer ☐ Prescribe only - Other cadre: …………. ☐Administer only ☐Prescribe and administer ☐ Prescribe only   *Please specify (e.g. auxiliary midwives/nurses)* |
| 3.5 Describe the supervision and/or mentoring guidelines and systems in place to ensure that every women who need antenatal corticosteroids, receives them. |
| **Please provide a summary of key bottlenecks.** |
| **After responding to the questions above, please make an overall assessment of whether the health workforce for the prevention and management of preterm birth, including the use of antenatal corticosteroids, is:**   - **Good** *(not a bottleneck to scale up)* - **Needs some improvements** *(minor bottleneck to scale up)* - **Needs major improvements** *(significant bottleneck to scale up)* - **Inadequate** *(very major bottleneck to scale up)* |
| **4. Essential medical products and technologies** |
| 4.1Indicate whether the following corticosteroids are included in the National Essential Medicines List (NEML) for the indication of foetal lung maturation in the context of preterm birth. If not, please explain why.  ☐ Dexamethasone  ☐ Betamethasone  ☐ Other, please specify |
| 4.2 If the use of antenatal corticosteroids for foetal lung maturation is included in the NEML, please specify the level of care:   - Primary level - First referral (secondary) level - Second referral (tertiary level), e.g. only where specialized OBGYN care is available |
| 4.3 Explain if sufficient medical products containing corticosteroids (branded or generic) that can be used for foetal lung maturation during the antenatal period to satisfy the need are licensed. If not, please explain why. |
| 4.4 Describe the national or local systems in place to accurately forecast the need for antenatal corticosteroids. If none exist, please explain why. |
| 4.5 Describe any indications of shortfalls in availability of antenatal corticosteroids at the national and sub-national level during the last 12 months. |
| **Please provide a summary of key bottlenecks.** |
| **After responding to the questions above, please make an overall assessment of whether the availability of antenatal corticosteroids for foetal lung maturation in management of preterm birth is:**   - **Good** *(not a bottleneck to scale up)* - **Needs some improvements** *(minor bottleneck to scale up)* - **Needs major improvements** *(significant bottleneck to scale up)* - **Inadequate** *(very major bottleneck to scale up)* |
| **5. Health service delivery** |
| 5.1 Describe the systems in place to promote the adherence to national standard treatment guidelines or clinical protocols on the use of antenatal corticosteroids for foetal lung maturation. If none exist, please explain why. For example:   - Describe the quality improvement programmes aiming at increasing use of antenatal corticosteroids. - Describe any specific efforts for private (for-profit) health care facilities. |
| 5.2 Explain whether the lowest level of care where antenatal corticosteroids for foetal lung maturation are administered is adequate. For example:   - Is it available at primary level? - Describe if multiple referrals lead to unnecessary delays. - Explain whether timely referrals to the level of care where antenatal corticosteroids are given are feasible. |
| 5.3 Explain if the organization of health care within facilities prevents women from receiving antenatal corticosteroids, when they need them. For example:   - Are there long waiting times or inappropriate opening hours at the level of care where the intervention is available, leading to low pick-up of antenatal corticosteroids? - Is the responsibility for identifying the woman in need, for prescribing and for administrating antenatal corticosteroids very fragmented? - Are there intra-service delays? |
| 5.4 Describe other barriers to the use of antenatal corticosteroids for foetal lung maturation that need to be addressed. For example:   - Attitudes of health care workers affecting the adoption of this intervention - Marital consent |
| **Please provide a summary of key bottlenecks.** |
| **After responding to the questions above, please make an overall assessment of whether the health service delivery for the prevention and management of preterm birth, including the use of antenatal corticosteroids, is:**   - **Good** *(not a bottleneck to scale up)* - **Needs some improvements** *(minor bottleneck to scale up)* - **Needs major improvements** *(significant bottleneck to scale up)* - **Inadequate** *(very major bottleneck to scale up)* |
| **6. Health information systems** |
| 6.1 Describe the information available on the use of antenatal corticosteroids for foetal lung maturation. For example:   - Describe the indicators used to assess the coverage of the use of antenatal corticosteroids. Please specify the reference document for the indicators used, including publication dates and the page. - Specify any information on the use of antenatal corticosteroids included in the HMIS. |
| 6.2 Explain if the use of antenatal corticosteroids is included in standard clinical records or checklists. For example:   - Birth records - Partographs - Safe childbirth checklists |
| 6.3 Explain if the critical review of the appropriate use of antenatal corticosteroids is included in protocols for clinical audits and perinatal death reviews. If not, please explain why. |
| **Please provide a summary of key bottlenecks.** |
| **After responding to the questions above, please make an overall assessment of whether the health information system for the prevention and management of preterm birth, including the use of antenatal corticosteroids, is:**   - **Good** *(not a bottleneck to scale up)* - **Needs some improvements** *(minor bottleneck to scale up)* - **Needs major improvements** *(significant bottleneck to scale up)* - **Inadequate** *(very major bottleneck to scale up)* |
| **7. Community ownership and partnership** |
| 7.1 Describe any specific efforts to increase the awareness of the general public, adolescent girls, pregnant women and young couples of the benefits of antenatal care, the prevention of preterm birth, and timely care seeking in health facility. :   - Are there IEC materials available in appropriate local language? - Describe the male involvement in maternal and newborn health issues. |
| - 1. Describe other challenges faced by women that limit the use of antenatal services. - Are antenatal services affordable to poorer families? - Are adequate transportation options to medical care facilities available? - How much knowledge do women have on this topic? Do they know their rights? - Are gender issues or socio-cultural factors barriers to use of services? |
| 7.3 Describe the level of engagement of the communities to increase the use of MNH services. For example:   - Explain whether community perspectives on health care have been taken into account. - Describe ways that community perspectives could be helpful in mobilizing and educating the community. |
| **Please provide a summary of key bottlenecks.** |
| **After responding to the questions above, please make an overall assessment of whether community ownership and participation for the prevention and management of preterm birth, including the use of antenatal corticosteroids, is:**   - **Good** *(not a bottleneck to scale up)* - **Needs some improvements** *(minor bottleneck to scale up)* - **Needs major improvements** *(significant bottleneck to scale up)* - **Inadequate** *(very major bottleneck to scale up)* |

**IDENTIFICATION OF SOLUTIONS** **TO ADDRESS THE CHALLENGES**

*Please add sheets as appropriate*

| **Intervention 1: *MANAGEMENT OF PRETERM BIRTH - Focus on antenatal corticosteroids*** | | |
| --- | --- | --- |
| **Summary of key bottlenecks *by order of priority*** | | Strategies and solutions to address identified challenges and bottlenecks |
| *Building block* | Priority bottlenecks |  |
| *Leadership and Governance* |  |  |
| *Health Finance* |  |  |
| *Health Workforce* |  |  |
| *Essential Medical products and Technologies* |  |  |
| *Health Service Delivery* |  |  |
| *Health Information Systems* |  |  |
| *Community Ownership and Participation* |  |  |

**Intervention 2: *SKILLED CARE AT BIRTH***

***Focus on the use of the partograph***

Appropriate care at birth should be provided by skilled personnel. A skilled attendant is an accredited health professional – such as a midwife, doctor or nurse – who has been educated and trained to proficiency in the skills needed to manage normal (uncomplicated) pregnancies, childbirth and the immediate postnatal period, and in the identification, management and referral of complications in women and newborns. For this exercise, we will focus on care needed during childbirth and the first 24 hours. Partograph, oxytocin, and clean birth kits/delivery sets are essential commodities for skilled care, but the tracer commodity that will be used for this exercise is the partograph.

| **1. Leadership and governance** |
| --- |
| 1.1 Explain whether skilled care at birth has been identified as a priority intervention package in the national RMNCH plan/strategy (specify the name of the documents). Explain if the plan/strategy includes specific actions to enable all pregnant women to access services.  If not included, please explain why. |
| 1.2 Describe the national standard treatment guidelines or clinical protocols covering skilled care at birth. Please specify the name of the guidelines/clinical protocol document and year of publication. If none exist, please explain why.  Do they include the following:   - Labour monitoring with the use of the partograph? - Clean delivery practices? - Detection of complications and timely referral for care? |
| 1.3Explain if skilled care and the use of partograph are recommended at all levels of care. Please specify:   - Community level (home delivery with a skilled birth attendant) - First level - Referral level |

| **Please provide a summary of the key bottlenecks.** |
| --- |
| **After responding to the questions above, please make an overall assessment of whether leadership and governance for skilled care at birth, including the use of partograph, is:**   - **Good** *(not a bottleneck to scale up)* - **Needs some improvements** *(minor bottleneck to scale up)* - **Needs major improvements** *(significant bottleneck to scale up)* - **Inadequate** *(very major bottleneck to scale up)* |

| **2. Health financing** |
| --- |
| 2.1Describe the costed RMNCH strategy that specifically includes an investment plan in the expansion of skilled care at birth. If one does not exist, please explain why. |
| 2.2 Describe the results-based financing mechanism in place to increase access to skilled care at birth to the most in need (poorest). Please briefly describe the mechanism, what is covered, and mention regions or districts where this is in place. If one does not exist, please explain why. |
| 2.3 Describe any financial barriers that prevents women from accessing/using skilled care at birth  For example:   - Describe any out-of-pocket payments that pose a barrier to skilled care for all women. - Describe if and how user fees represent a barrier to skilled care of pregnant women*.* |

| **Please provide a summary of the key bottlenecks.** |
| --- |
| **After responding to the questions above, please make an overall assessment of whether health financing for skilled care at birth, including the use of partograph, is:**   - **Good** *(not a bottleneck to scale up)* - **Needs some improvements** *(minor bottleneck to scale up)* - **Needs major improvements** *(significant bottleneck to scale up)* - **Inadequate** *(very major bottleneck to scale up)* |

| **3. Health workforce** |
| --- |
| 3.1 Explain if there are sufficient numbers of trained health care workers to provide services at each level of care where skilled delivery care should be provided. Issues may include:   - There are not sufficient midwifery personnel, who can provide skilled care at birth, including the use of partograph - There might be sufficient numbers of motivated health care workers, but they do not have the necessary competencies to use the partograph - There may be sufficient numbers of midwifery personnel in the country but they are not deployed to remote areas   Please indicate the ratios of midwifery personnel per population density nationally and in the most deprived areas. |
| 3. 2 Explain whether there are (standard) job descriptions for midwifery personnel at all levels of care. Please explain how they articulate the responsibilities of staff in relation to the provision of skilled delivery care. If none exists, please explain why. |
| 3.3 Describe the competency-based training programmes for midwives and explain how they are organized. If none exist, please explain why |
| 3.4Explain if there are supervision and/or mentoring guidelines and systems in place to ensure that women receive quality delivery care (e.g. supervision on the use of the partograph). *Please explain how these are implemented.* |
| **Please provide a summary of the key bottlenecks.** |
| **After responding to the questions above, please make an overall assessment of whether the health workforce for skilled care at birth, including the use of partograph, is:**   - **Good** *(not a bottleneck to scale up)* - **Needs some improvements** *(minor bottleneck to scale up)* - **Needs major improvements** *(significant bottleneck to scale up)* - **Inadequate** *(very major bottleneck to scale up)* |
| **4. Essential medical products and technologies** |
| 4.1 Describe and specify the mechanism of national/district procurement and distribution of the partograph in health facilities and whether it is functional. If one does not exist, please explain why. |
| 4.2 Describe and explain the reasons for stock-outs of partographs reported at the national/subnational level in the last 12 months, if any. |
| 4.3 List additional challenges related to the distribution and use of partograph. |
| **Please provide a summary of the key bottlenecks.** |
| **After responding to the questions above, please make an overall assessment of whether PSM of partograph for skilled care at birth is:**   - **Good** *(not a bottleneck to scale up)* - **Needs some improvements** *(minor bottleneck to scale up)* - **Needs major improvements** *(significant bottleneck to scale up)* - **Inadequate** *(very major bottleneck to scale up)* |
| **5. Health service delivery** |
| 5.1 Please examine the challenges that may be faced by the health system to deliver/reach women and newborns with services (coverage issues). |
| 5.1.1 Describe the public-private partnerships in place to enhance service delivery. Please specify the proportion of skilled birth attendants provided by the public and private sectors. |
| 5.1.2 Explain whether there are sufficient facilities providing skilled delivery services (hospitals, health centres, birthing centres), especially in rural and remote areas. Please review and provide a breakdown of facilities per population density including the remote and rural areas. |
| *5.1.3* Explain whether skilled delivery services are easily accessible to women, especially those in remote areas. Please consider physical access to services in urban and rural areas and highlight some of the access issues below. |
| - - 1. Explain whether skilled delivery services are provided 24 hours, 7 days a week at all levels of care: - In most urban areas? - In most rural areas?   Please summarize some of the challenges to the timing of the provision of services. |
| - - 1. Explain whether most skilled delivery facilities, particularly in rural areas, have adequate referral systems in place. If not, please explain why. |
| 5.1.6 Explain if health facilities that provide services in urban areas generally have reliable electricity and if the national electricity grid extends to rural and remote areas. |
| 5.2 Please examine challenges to the health system to deliver quality services to all women and newborns. For example, are there quality improvement mechanisms in place for skilled delivery services (e.g. supervision and mentorship programmes, use of checklists, job-aids, periodic service reviews)? |
| **Please provide a summary of the key bottlenecks.** |
| **After responding to the questions above, please make an overall assessment of whether health service delivery for skilled care at birth, including the use of partograph, is:**   - **Good** *(not a bottleneck to scale up)* - **Needs some improvements** *(minor bottleneck to scale up)* - **Needs major improvements** *(significant bottleneck to scale up)* - **Inadequate** *(very major bottleneck to scale up)* |
| **6. Health information systems** |
| 6.1Explain if the country has defined indicators in the national strategy/plan to assess the coverage of skilled care at birth. Please specify the indicators and the page in the document. If none exist, please explain why. |
| - 1. Explain whether indicators measuring skilled care at birth are integrated and collected through routine HMIS and describe the indicators used. |
| - 1. Describe whether the data on skilled care at birth is collected and used by health workers themselves to improve quality and performance at the facility level. Describe how the data is used? |
| **Please provide a summary of the key bottlenecks.** |
| **After responding to the questions above, please make an overall assessment of whether health information system for skilled care at birth, including the use of partograph, is:**   - **Good** *(not a bottleneck to scale up)* - **Needs some improvements** *(minor bottleneck to scale up)* - **Needs major improvements** *(significant bottleneck to scale up)* - **Inadequate** *(very major bottleneck to scale up)* |
| **7. Community ownership and partnership** |
| 7.1 Describe specific efforts made to increase the awareness of the general public, adolescent girls, pregnant women and young couples of the benefits of skilled care at birth in the health facility   - Are there IEC materials available in appropriate local language? - Describe male involvement in facilitating the use of childbirth services? |
| - 1. Describe other challenges faced by women that limit the use of childbirth and skilled care at birth. - Is skilled care affordable to poorer families? - Are adequate transportation options to medical care facilities available? - How much knowledge do women have on this topic? Do they know their rights? - Are gender issues or socio-cultural factors barriers to use skilled care? |
| 7.3 Describe the level of engagement of the communities to increase the use of skilled care at birth. For example:   - Explain if community perspectives on health care been taken into account and if community representatives are involved in audit committees and reviews. - Describe ways community perspectives could be helpful in mobilizing and educating the community. |
| **Please provide a summary of the key bottlenecks.** |
| **After responding to the questions above, please make an overall assessment of whether community ownership and participation for skilled care at birth, including the use of partograph, is:**   - **Good** *(not a bottleneck to scale up)* - **Needs some improvements** *(minor bottleneck to scale up)* - **Needs major improvements** *(significant bottleneck to scale up)* - **Inadequate** *(very major bottleneck to scale up)* |

**IDENTIFICATION OF SOLUTIONS** **TO ADDRESS THE CHALLENGES**

*Please add sheets as appropriate*

| **Intervention 2: *SKILLED CARE AT BIRTH - Focus on the use of the partograph*** | | |
| --- | --- | --- |
| **Summary of key bottlenecks *by order of priority*** | | Strategies and solutions to address identified challenges and bottlenecks |
| *Building block* | *Priority bottlenecks* |  |
| *Leadership and Governance* |  |  |
| *Health Finance* |  |  |
| *Health Workforce* |  |  |
| *Essential Medical products and Technologies* |  |  |
| *Health Service Delivery* |  |  |
| *Health Information Systems* |  |  |
| *Community Ownership and Participation* |  |  |

**Intervention 3: *BEmOC***

***Tracer: focus on “assisted vaginal delivery”***

Basic emergency obstetric care (BEmOC) includes seven signal functions to be performed in health centres without the need for an operating theatre. For the purpose of this exercise, “assisted vaginal delivery” has been selected as a “tracer” signal function to assess challenges related to the delivery of BEmOC services. Operative vaginal delivery refers to the application of either a vacuum device or forceps to assist the mother in effecting vaginal delivery of a foetus.

| **1. Leadership and governance** |
| --- |
| 1.1 Explain whether the RMNCH plan/strategy includes specific actions for scaling-up BEmOC to perform assisted vaginal delivery. If not, please explain why.  In addition, explain if BEmOC, including assisted vaginal delivery is in the basic/essential health care package. If not, please explain why. |
| 1.2 Describe the national **RMNCH policies** in place to promote safe motherhood in general and BEmOC in particular. (If none exists, please explain why.)   - Explain if policies explicitly mention the rights of patients. - Explain if BEmOC is addressed in multi-agency processes such as sector-wide approaches and Poverty Reduction Strategy Papers. |
| 1.3 Explain if there are national standard treatment guidelines or clinical protocols on assisted vaginal delivery as part of the BEmOC package. Please specify name of the document and year of publication. If they do not exist, please explain why.   - Explain if they specify key actions for the use of oxytocin and vacuum extractor/forceps. - Explain if they provide guidelines for management and/or referral of complications due to assisted vaginal delivery. - Explain if all recommendations are regularly updated and in line with current best practices (e.g. latest WHO guidelines). |
| 1.4 Describe the level of care that assisted vaginal delivery as part of the BEmOC package is recommended. Please, specify reference document.   - First level /outreach - Referral level (secondary and tertiary) |
| **Please provide a summary of key bottlenecks.** |
| **After responding to the questions above, please make an overall assessment of whether leadership and governance mechanisms in place for assisted vaginal delivery as part of BEmOC is:**   - **Good** *(not a bottleneck to scale up)* - **Needs some improvements** *(minor bottleneck to scale up)* - **Needs major improvements** *(significant bottleneck to scale up)* - **Inadequate** *(very major bottleneck to scale up)* |
| **2. Health financing** |
| 2.1 Describe any specific financing issues related to the expansion of BEmOC services to perform assisted vaginal delivery at all recommended levels of care. For example:  Describe whether in centralized procurement and distribution systems, funds are sufficient to maintain BEmOC services to perform assisted vaginal delivery or to procure and distribute necessary equipment (oxytocin, vacuum extractors for example) at all recommended levels of care for all women in need and at district level in decentralized systems.. |
| 2.2 Describe any financial barriers that prevent women from using BEmOC facilities that can perform assisted vaginal delivery. For example:   - Out-of-pocket payments as barriers to care-seeking for all women - User fees as barriers to admission of pregnant women - Other financial barriers (describe) |
| **Please provide a summary of key bottlenecks.** |
| **After responding to the questions above, please make an overall assessment of whether health financing for BEmOC to perform assisted vaginal delivery for all women is:**   - **Good** *(not a bottleneck to scale up)* - **Needs some improvements** *(minor bottleneck to scale up)* - **Needs major improvements** *(significant bottleneck to scale up)* - **Inadequate** *(very major bottleneck to scale up)* |
| **3. Health workforce** |
| 3.1 Explain whether there are sufficient numbers of competent health care workers who can perform assisted vaginal delivery, at each level of care, where this intervention should be implemented. Issues may include:   - There is insufficient skilled health workers that are able to perform assisted vaginal delivery in the health facilities - There might be sufficient numbers of trained health care workers, but they do not have the necessary competencies to perform assisted vaginal delivery - How is the distribution of personnel trained to perform assisted vaginal delivery between rural and urban populations? Is there a plan for placement of professionals in underserved areas*?* |
| 3.2 What cadre of health care workers are trained/authorized to perform assisted vaginal delivery using vacuum extractors or forceps at BEmOC facilities?   - Midwives - Auxiliary midwives - Nurses - Auxiliary nurses: - Physicians/clinicians - Specialists (OB-G) - Other cadres (please specify all): ………. |
| 3.3 What cadre of health care workers are authorized to prescribe and/or administer oxytocin during child birth?   - Midwives ☐Administer only ☐Prescribe and administer ☐ Prescribe only - Auxiliary midwives: ☐Administer only ☐Prescribe and administer ☐ Prescribe only - Nurses: ☐Administer only ☐Prescribe and administer ☐ Prescribe only - Specialists (OB-G): ☐Administer only ☐Prescribe and administer ☐ Prescribe only - Physicians/clinicians ☐Administer only ☐Prescribe and administer ☐ Prescribe only - Other cadre (please specify all): ……… ☐Administer only ☐Prescribe and administer ☐ Prescribe only |
| 3. 4 Explain the whether there are job description and job aids for health workers at all levels of care, which reflect their role in performing assisted vaginal delivery at BEmOC facilities. Explain whether the manuals detailing standards of practice are available and accessible for staff use. |
| 3.5 Are there competency-based training programmes through which the respective cadre of health care workers listed below acquire the necessary knowledge and skills to perform assisted vaginal delivery and use oxytocin during delivery?   - Nurses ☐Pre-service training ☐ In-service training - Midwives ☐Pre-service training ☐ In-service training - Auxiliary midwives: ☐Pre-service training ☐ In-service training - Clinical officers ☐Pre-service training ☐ In-service training - Physicians/clinicians ☐Pre-service training ☐ In-service training - Specialists (OB-G) ☐Pre-service training ☐ In-service training - Other cadre: …………. ☐Pre-service training ☐ In-service training |
| 3.6 Describe the supervision and mentoring mechanisms is in place to ensure all health workers that provide BEmOC services maintain their competency to perform assisted vaginal delivery and use oxytocin during delivery as per national guidelines. |
| **Please provide a summary of key bottlenecks.** |
| **After responding to the questions above, please make an overall assessment of whether the health work force responsible for performing assisted vaginal delivery for all women at BEmOC facilities is:**   - **Good** *(not a bottleneck to scale up)* - **Needs some improvements** *(minor bottleneck to scale up)* - **Needs major improvements** *(significant bottleneck to scale up)* - **Inadequate** *(very major bottleneck to scale up)* |
| **4. Essential medical products and technologies** |
| 4.1 Explain if the country has a national essential devices list and if the vacuum extractor and forceps are included in the National Essential Devices List for the indication of assisted vaginal delivery. If a national essential device list does not exist, please explain why.  Explain if oxytocin is included in the National Essential Medical List (NEML) for the indication of child birth. |
| 4.2 Explain if sufficient medical products (branded or generic) that can be used as uterotonics for child birth are licensed. |
| 4.3 Describe functional national or local systems in place to forecast accurately and distribute the following essential products when needed:   - Oxytocin - Vacuum extractors - Forceps |
| 4.4 Describe and explain the causes for stock-outs of oxytocin at national and/or sub-national levels in the last twelve months, if any.  Describe the functional logistics information system able to assess adequate supplies and to detect the need for vacuum extractors/forceps in BEmOC facilities in need of the device, if available. |
| 4.5 Describe additional challenges related to the procurement and distribution of oxytocin and vacuum extractor in BEmOC facilities. |
| **Please provide a summary of key bottlenecks.** |
| **After responding to the questions above, please make an overall assessment whether the procurement and supply management system to perform assisted vaginal delivery for all women at BEmOC facilities is:**   - **Good** *(not a bottleneck to scale up)* - **Needs some improvements** *(minor bottleneck to scale up)* - **Needs major improvements** *(significant bottleneck to scale up)* - **Inadequate** *(very major bottleneck to scale up)* |
| **5. Health service delivery** |
| 5.1 Explain if there are sufficient health facilities at all levels organized and equipped to perform assisted vaginal delivery in line with national guidelines, particularly at the lowest level.   - What types of health facilities perform more of this signal function? - Explain if the BEmOC facilities are adequate in terms of geographic distribution and numbers and when these services were last mapped. - Explain if there is a system in place for regular room by room inspection to ensure that equipment and supplies needed for BEmOC are present and functioning. If not, please explain why. - Explain if there is a management system in place to ensure that BEmOC is available 24 hours a day. If not, please explain why. - Explain if health facilities are maintained at standards to ensure quality care for women and if they are in line with national guidelines. |
| 5.2 Describe the systems in place to promote the adherence to national standard and clinical protocols on basic newborn care (see section 1.2 on leadership and governance for relevant guidelines).For example:   - Explain whether there are quality improvement mechanisms in place with standardized tools such as check lists to assess and enhance the quality of services provided to women. - Explain if there are specific efforts made by public and private (for-profit) health care facilities to promote and ensure quality BEmOC services in general. - Explain if assisted vaginal delivery, as part of BEmOC signal functions, is performed regularly and assessed every three to six months in health facilities. - Explain if manuals detailing standards of practice are available and accessible for staff use. - Describe if clinical audits are conducted. If not, please explain why. - Identify the percentage of BEmOC facilities certified. |
| 5.3Explain if there is a functional referral system to strengthen linkages between health facilities and the communities. If not, please explain why.  Describe the communication systems in place between hospitals and ambulances. Are ambulances services functional and effective? Are communication systems (radios/phones) available for referrals? |
| 5.4 Describe other health service delivery barriers to the performance of assisted vaginal delivery at BEmOC facilities, which need to be addressed. |
| **Please provide a summary of key bottlenecks.** |
| **After responding to the questions above, please make an overall assessment of whether health service delivery to allow BEmOC facilities to perform assisted vaginal delivery is:**   - **Good** *(not a bottleneck to scale up)* - **Needs some improvements** *(minor bottleneck to scale up)* - **Needs major improvements** *(significant bottleneck to scale up)* - **Inadequate** *(very major bottleneck to scale up)* |
| **6. Health information systems** |
| 6.1Describe the information available on assisted vaginal delivery at BEmOC facilities. Describe indicators used by the routine system to monitor BEmOC services For example:   - Describe the indicators used to assess BEmOC services and explain if data are collected in accordance with EmOC-related indicators: Met need for BEmOC - Explain if any information is available on the number of assisted vaginal delivery performed monthly or proportion of all births in BEmOC facilities, etc. and if these indicators are routinely collected. - Explain and specify if any information on the use of oxytocin during child birth is available in the HMIS. |
| 6.2 Explain how the system collects data on case fatality rates and if the critical review of the quality of assisted vaginal delivery is included in protocols for maternal deaths audits and perinatal death reviews. What is the difference in fatality rates between BEmOC public and private hospitals? What could be the reasons? |
| 6.3 Describe the completeness and quality of facility records. |
| **Please provide a summary of key bottlenecks.** |
| **After responding to the questions above, please make an overall assessment of whether health information system for BEmOC including assisted vaginal delivery as a signal function is:**   - **Good** *(not a bottleneck to scale up)* - **Needs some improvements** *(minor bottleneck to scale up)* - **Needs major improvements** *(significant bottleneck to scale up)* - **Inadequate** *(very major bottleneck to scale up)* |
| **7. Community ownership and partnership** |
| 7.1 Describe any specific efforts to increase the awareness of the generic public, adolescent girls, pregnant women and young couples of the benefits of timely utilization of BEmOC in health facilities. For example:   - Are there IEC materials available in appropriate local language? - Describe male involvement in MNH issues and support the use of BEmOC services. |
| - 1. Describe other challenges faced by women that limit the use of BEmOC services - Is BEmOC affordable to poorer families? - Are adequate transportation options to medical care facilities available? - Do women have knowledge on the timely use of BEmOC services? Do they know their rights? - Are gender issues or socio-cultural factors barriers to use of services? |
| 7.3 Describe the level of engagement of the communities to increase the use of BEmOC services? For example:   - Explain if community perspectives on health care been taken into account and if community representatives are involved in audit committees and reviews. - Describe what ways they could be helpful in mobilizing and educating the community. |
| **Please provide a summary of key bottlenecks.** |
| **After responding to the questions above, please make an overall assessment of whether community ownership and partnership to enhance the use of BEmOC services is:**   - **Good** *(not a bottleneck to scale up)* - **Needs some improvements** *(minor bottleneck to scale up)* - **Needs major improvements** *(significant bottleneck to scale up)* - **Inadequate** *(very major bottleneck to scale up)* |

**IDENTIFICATION OF SOLUTIONS** **TO ADDRESS THE CHALLENGES**

*Please add sheets as appropriate*

| **Intervention 3: *BEmOC - Focus on assisted vaginal delivery*** | | |
| --- | --- | --- |
| **Summary of key bottlenecks *by order of priority*** | | Strategies and solutions to address identified challenges and bottlenecks |
| *Building block* | *Priority bottlenecks* |  |
| *Leadership and Governance* |  |  |
| *Health Finance* |  |  |
| *Health Workforce* |  |  |
| *Essential Medical products and Technologies* |  |  |
| *Health Service Delivery* |  |  |
| *Health Information Systems* |  |  |
| *Community Ownership and Participation* |  |  |

**Intervention 4: *CEmOC***

***Tracer: focus on caesarean section and blood transfusion***

Comprehensive emergency obstetric and newborn care (CEmOC), typically delivered in district hospitals, includes all functions in BEmOC, plus caesarean section and safe blood transfusion. For the purpose of this exercise, ‘Caesarean section’ and ‘blood transfusions’ have been selected as “tracer” signal function to assess challenges related to the delivery of CEmOC services.

| **1. Leadership and governance** |
| --- |
| 1.1Explain whether the national RMNCH plan/strategy includes specific actions for scaling-up CEmOC. If not, please explain why. |
| 1.2 Explain if CEmOC is addressed in multi-agency processes such as sector-wide approaches and Poverty Reduction Strategy Papers. If not, please explain why. |
| 1.3 Describe the national standard treatment guidelines or clinical protocols on caesarean section and blood transfusions as part of the CEmOC package. Please address the presence and describe the following:   - Guidelines for providing blood transfusions to women in labour. - Guidelines for the specific indications for caesarean section. - Are all recommendations regularly updated and in line with current best practices (e.g. latest WHO guidelines)? |
| 1.4 Explain if all relevant policies or regulations are aligned with the recommendations for the provision of blood transfusions or caesarean section for all women in need. For example:   - Explain whether staffing standards for facilities are aligned with CEmOC coverage plans. - Explain whether plans for blood banks are aligned to ensure availability at all CEmOC facilities. |
| **Please provide a summary of key bottlenecks.** |
| **After responding to the questions above, please make an overall assessment of whether leadership and governance mechanisms in place for caesarean sections as part of CEmOC is:**   - **Good** *(not a bottleneck to scale up)* - **Needs some improvements** *(minor bottleneck to scale up)* - **Needs major improvements** *(significant bottleneck to scale up)* - **Inadequate** *(very major bottleneck to scale up)* |
| **2. Health financing** |
| 2.1 Describe specific financing issues related to the expansion of CEmOC services to perform blood transfusions and caesarean sections at recommended levels of care. For example:   - *At the national level:*   In centralized procurement and distribution systems, are funds sufficient to maintain CEmOC services at all recommended levels of care for all women in need in all health facilities where they are required for  - Blood transfusions?  - Caesarean sections?   - *Explain if there are sufficient funds for services at the district or facility level.* |
| 2.2 Describe any financial barriers that prevent women from using CEmOC facilities. For example:   - *Describe any out-of-pocket payments that pose a barrier to care-seeking for all women.* - *Describe if and how user fees represent a barrier to admission of pregnant women.* |
| 2.3 Describe other financial barriers to the expansion of CEmOC facilities that can perform caesarean sections or provide blood transfusions as recommended. |
| **Please provide a summary of key bottlenecks.** |
| **After responding to the questions above, please make an overall assessment of whether health financing is in place for caesarean section as part of CEmOC is:**   - **Good** *(not a bottleneck to scale up)* - **Needs some improvements** *(minor bottleneck to scale up)* - **Needs major improvements** *(significant bottleneck to scale up)* - **Inadequate** *(very major bottleneck to scale up)* |
| **3. Health workforce** |
| 3.1 Explain if there are sufficient numbers of competent health care workers who can provide CEmOC where it should be implemented. Issues may include:   - There are insufficient skilled health workers that are able to perform caesarean sections and blood transfusions in the health facilities - There might be sufficient numbers of trained health care workers, but they do not have the necessary competencies to perform interventions - What is the distribution of personnel trained to provide CEmOC between rural and urban populations? Is there a plan for placement of professionals in underserved areas? |
| 3.2 What cadre of health care workers are trained to perform caesarean section at CEmOC health facilities?   - Midwives - Auxiliary midwives - Nurses - Clinical Officers - Physicians/clinicians - Other cadre (please specify all): ………. |
| 3.3 What cadre of health care workers are authorized to provide blood transfusions at CEmOC health facilities?   - Midwives - Auxiliary midwives - Nurses - Clinical Officers - Physicians/clinicians - Other cadre (please specify all): |
| 3. 4 Explain whether there are job descriptions and job aids for health workers at all levels of care which reflect their role in performing caesarean sections in CEmOC facilities; and if manuals detailing standards of practice are available and accessible for staff use. |
| 3.5 Are there competency-based training programmes through which the respective cadre of health care workers acquire the necessary knowledge and skills to perform caesarean sections?   - Nurses ☐Pre-service training ☐ In-service training - Midwives ☐Pre-service training ☐ In-service training - Auxiliary midwives: ☐Pre-service training ☐ In-service training - Clinical officers ☐Pre-service training ☐ In-service training - Physicians/clinicians ☐Pre-service training ☐ In-service training - Specialists (OB-G) ☐Pre-service training ☐ In-service training - Other cadre: …………. ☐Pre-service training ☐ In-service training |
| 3.6 Describe the supervision and mentoring mechanism in place to ensure that all health workers that provide CEmOC services maintain their competency to perform caesarean sections and blood transfusions as per national guidelines. If not, please explain why. |
| **Please provide a summary of key bottlenecks.** |
| **After responding to the questions above, please make an overall assessment of whether the health work force to perform caesarean sections as part of CEmOC facilities is:**   - **Good** *(not a bottleneck to scale up)* - **Needs some improvements** *(minor bottleneck to scale up)* - **Needs major improvements** *(significant bottleneck to scale up)* - **Inadequate** *(very major bottleneck to scale up)* |
| **4. Essential medical products and technologies** |
| 4.1 Explain if the systems in place to ensure adequate supplies of:   - Safe blood transfusions for women in labour who need it. Please describe the mechanisms or the bottlenecks. - Surgical kits for caesarean sections for women in labour who need it. Please describe the mechanisms or the bottlenecks.   Explain if sufficient blood replacement products are licensed |
| 4.2 Explain if there are functional national or local systems in place to forecast accurately and distribute these essential products when needed. If not, please explain why. |
| 4.3 Describe and explain the root causes of the following stock-outs at national and sub-national level in the last twelve months, if any.   - Blood and blood products - Surgical kits |
| 4.4 Provide additional challenges related to the procurement and distribution of the following:   - Blood and blood products? - Surgical kits |
| **Please provide a summary of key bottlenecks.** |
| **After responding to the questions above, please make an overall assessment of whether the procurement and supply management system to perform blood transfusions and caesarean sections at CEmOC facilities is:**   - **Good** *(not a bottleneck to scale up)* - **Needs some improvements** *(minor bottleneck to scale up)* - **Needs major improvements** *(significant bottleneck to scale up)* - **Inadequate** *(very major bottleneck to scale up)* |
| **5. Health service delivery** |
| 5.1 Explain if there are CEmOC facilities with appropriate human resource skills to perform caesarean sections and blood transfusions available to all women, including for those living in remote areas? Address the following:   - What types of health facilities perform CEmOC? - Are the CEmOC facilities adequate in terms of geographic distribution and numbers (1 comprehensive per 500,000 people)? - When were these services mapped? - Is a management system in place to ensure that CEmOC is available 24 hours a day |
| 5.2 Describe the systems in place to promote adherence to national standards and clinical protocols on CEmOC. For example:   - Describe quality improvement mechanisms in place with standardized tools such as checklists to assess and enhance the quality of services provided to women. - Explain specific efforts made by public and private (for-profit) health care facilities to promote and ensure quality CEmOC services in general. - Explain if caesarean sections, as part of CEmOC signal functions, are performed regularly and if are assessed every three to six months in health facilities. - Explain whether manuals detailing standards of practice are available and accessible for staff use. - Are clinical audits conducted? - Explain if a room by room inspection is performed regularly to ensure that equipment and supplies needed for CEmOC is present and functioning and if infection prevention measures are in place. If not, please explain why. |
| 5.3 Explain the difference in caesarean sections performance rates in CEmOC   - Between public and private hospitals (identify the reasons) - Between rural and urban areas (identify the reasons) |
| - 1. Describe the difference in fatality rates between CEmOC public and private hospitals? What could be the reasons? |
| 5.5 Explain if there is a functional referral system to strengthen linkages between health facilities and the community. If not, please explain why.   - Describe the communication systems in place between health hospitals and ambulances and explain if ambulance services are functional and effective. - Explain if communication systems (radios/phones) are available for referrals. |
| 5.6 Describe other barriers to the performance of caesarean sections or blood transfusions at CEmOC facilities that need to be addressed |
| **Please provide a summary of key bottlenecks.** |
| **After responding to the questions above, please make an overall assessment of whether health service delivery to allow CEmOC facilities to perform blood transfusion and caesarean section is:**   - **Good** *(not a bottleneck to scale up)* - **Needs some improvements** *(minor bottleneck to scale up)* - **Needs major improvements** *(significant bottleneck to scale up)* - **Inadequate** *(very major bottleneck to scale up)* |
| **6. Health information systems** |
| 6.1Describe the information available on caesarean sections at CEmOC facilities and what indicators are used by the routine system to monitor CEmOC services. For example:   - Describe the indicators used to assess CEmOC services and explain if data are collected in accordance with EmOC-related indicators: Met need for CEmOC. - Explain if any information is available on the number of caesarean sections performed monthly or proportion of all births in CEmOC facilities, etc. and if these indicators are routinely collected. - Explain and specify if any information is available in HMIS on the use of blood transfusions during child birth available in HMIS. |
| - 1. Explain if the system that collects data on the review of the quality of caesarean sections is included in protocols for maternal death audits and perinatal death reviews. |
| - 1. What is the completeness and quality of facility records?   Are maternal and newborn facility records analysed for completeness? If yes, what percentage of records are complete?  Describe the information on quality of the data that is included in facility records |
| **Please provide a summary of key bottlenecks.** |
| **After responding to the questions above, please make an overall assessment of whether monitoring and evaluation of CEmOC including assisted caesarean sections and blood transfusions as signal functions is:**   - **Good** *(not a bottleneck to scale up)* - **Needs some improvements** *(minor bottleneck to scale up)* - **Needs major improvements** *(significant bottleneck to scale up)* - **Inadequate** *(very major bottleneck to scale up)* |
| **7. Community ownership and partnership** |
| 7.1 Describe the challenges faced by women that limit the use of CEmOC services.   - Is CEmOC affordable to poorer families? - Are adequate transportation options to medical care facilities available? - How much knowledge do women have on this topic? Do they know their rights? - Are there gender issues or socio-cultural factors barriers to use of services? |
| 7.2 Describe the level of engagement of the communities to increase the use of CEmOC services. For example   - Explain if community perspectives on health care have been taken into account and if community representatives are involved in audit committees and reviews. - Describe ways in which they could be helpful in mobilizing and educating the community. |
| **Please provide a summary of key bottlenecks.** |
| **After responding to the questions above, please make an overall assessment of whether community ownership and partnership to enhance the use of CEmOC services is:**   - **Good** *(not a bottleneck to scale up)* - **Needs some improvements** *(minor bottleneck to scale up)* - **Needs major improvements** *(significant bottleneck to scale up)* - **Inadequate** *(very major bottleneck to scale up)* |

**IDENTIFICATION OF SOLUTIONS** **TO ADDRESS THE CHALLENGES**

*Please add sheets as appropriate*

| **Intervention 4: *CEmOC - Focus on caesarean section*** | | |
| --- | --- | --- |
| **Summary of key bottlenecks *by order of priority*** | | Strategies and solutions to address identified challenges and bottlenecks |
| *Building block* | *Priority bottlenecks* |  |
| *Leadership and Governance* |  |  |
| *Health Finance* |  |  |
| *Health Workforce* |  |  |
| *Essential Medical products and Technologies* |  |  |
| *Health Service Delivery* |  |  |
| *Health Information Systems* |  |  |
| *Community Ownership and Participation* |  |  |

**Intervention 5: *BASIC NEWBORN CARE FOR ALL NEWBORNS***

***Focus on cleanliness/cord care, warmth, and feeding support***

Basic newborn care (BNC) comprises of a set of basic preventive measures that are needed to ensure the survival of all newborns including assisting babies to breathe, if needed. BNC includes several key actions, but for the purpose of this exercise, we will focus on cleanliness, thermal control (including drying and wrapping, skin-to-skin, and delayed bathing), and support for breastfeeding.

| **1. Leadership and governance** |
| --- |
| 1.1 In the national RMNCH plan/strategy (specify the name of the documents), explain if basic newborn care (BNC) has been identified as a priority intervention, and if the plan/strategy includes specific actions for care and monitoring of all newborn babies, home-based care, and feeding support. If not, please explain why. |
| 1.2 Describe the national standard treatment guidelines or clinical protocols covering immediate care for the newborns after delivery, prevention and management of umbilical cord infections, basic care for the newborns during postnatal visits. (Specify the name of the guidelines/clinical protocol document and year of publication)   - Explain if they include recommendations on the delivery room temperature to prevent hypothermia. - Describe the key actions for care and monitoring of newborns related to warmth, hygiene, cord care, immediate exclusive breastfeeding counselling and support, support for alternative feeding methods, recognition of danger signs and care seeking, and timing of postnatal visits. - Describe the key actions for home-based care for newborns and explain whether the use of chlorhexidine for cord care at the community level is recommended. - Explain if all recommendations are regularly updated and in line with current best practices (e.g. latest WHO guidelines). |
| 1.3 At what level of care is basic newborn care for all newborns recommended?   - Community level - First level /outreach - Referral level (secondary and tertiary) |
| 1.4 Explain if the country implements the Baby-friendly Hospital Initiative (BFHI) and describe at what level is the initiative implemented.    Explain if the BFHI package has been extended to include “compliance with the International Code of Marketing of Breastmilk Substitutes”.  Explain if there is effective national legislation in place regulating the marketing of breastmilk substitutes, that early initiation and exclusive breastfeeding for the first month of life is promoted, protected and supported.  Explain whether policies and/or statements of relevant professional associations (e.g. midwives, paediatricians) support early initiation and exclusive breastfeeding for the first six months. |
| 1.5 Explain whether all relevant policies or regulations are aligned with the recommended basic newborn care for all newborns For example:   - Describe how health facilities are organized and equipped for appropriate care and monitoring of all newborns in line with national guidelines. - Describe how health facilities maintain hygiene standards to ensure quality care for newborns. - Explain whether all health facilities where babies are born comply with the criteria of the BFHI. - Explain if there is a cadre of health personnel authorized and trained to conduct postnatal home visits. - Explain if health workers that assess and manage newborns provide adequate feeding (including exclusive breastfeeding) counselling and support (more details in section on human resources). |
| **Please provide a summary of key bottlenecks** |
| **After responding to the questions above, please make an overall assessment of whether leadership and governance mechanisms in place for basic newborn care for all newborns is:**   - **Good** *(not a bottleneck to scale up)* - **Needs some improvements** *(minor bottleneck to scale up)* - **Needs major improvements** *(significant bottleneck to scale up)* - **Inadequate** *(very major bottleneck to scale up)* |
| **2. Health financing** |
| 2.1 Describe any specific financing issues related to the implementation of newborn care services at all recommended level of care For example:   - *At the national level:*   - Explain if funds are sufficient in centralized procurement and distribution systems to procure and distribute necessary equipment (weighing scale, thermometers, warmers, etc) at all recommended levels of care to provide basic newborn care to all newborns and if they are provided in a continuous and sustainable manner to all health facilities where they are required.      - *At the district or facility level:*   Explain if funds are sufficient in decentralized procurement and distribution systems to procure and distribute necessary equipment (weighing scale, thermometers, warmers, etc) at all recommended levels of care to provide basic newborn care to all newborns and if they are provided in a continuous and sustainable manner to all health facilities where they are required. |
| 2.2 Describe any financial barriers that prevent newborns from receiving appropriate care at the health facility and at home. For example:   - Describe any out-of-pocket payments that are a barrier to care-seeking for all sick newborns. - Describe user fees that represent a barrier to admission of sick newborns. - Explain if patients/families need to purchase chlorhexidine for cord care at home, because they are not provided by health facility. - Does the price of chlorhexidine is a financial barrier? - Explain if the costs related to extra-care pose an issue for the clients who need it.(e.g. fees for a neonatologist) |
| 2.3 Describe other financial barriers to the expansion of basic newborn care services for all newborns in all health facilities as recommended |
| **Please provide a summary of key bottlenecks.** |
| **After responding to the questions above, please make an overall assessment of whether health financing for basic newborn care for all newborns is:**   - **Good** *(not a bottleneck to scale up)* - **Needs some improvements** *(minor bottleneck to scale up)* - **Needs major improvements** *(significant bottleneck to scale up)* - **Inadequate** *(very major bottleneck to scale up)* |
| **3. Health workforce** |
| 3.1 Explain whether there are sufficient numbers of competent health care workers who can provide basic newborn care services, at each level of care, where this intervention should be implemented. Issues may include:   - There is insufficient health workers to provide basic newborn care and recognize newborns with danger signs in the health facilities and at home - There might be sufficient numbers of trained health care workers, but they do not have the necessary competencies for the assessment and recognition of newborns with danger signs? - There might be sufficient numbers of trained health care workers, but outreach services are not in place so that they can provide newborn care during home visits - How is the distribution of skilled personnel trained to provide BNC between rural and urban populations? Is there a human resource strategy to expand BNC to all newborns leaving in remote areas through community health workers? |
| 3.2 What cadre of health care workers are authorized and skilled to provide basic newborn care for all newborns?   - Community health workers: - Auxiliary nurse midwives: - Nurses / auxiliary nurses: - Midwives/ auxiliary midwives - Other cadre (please specify all): ……….   What cadre of health care workers are authorized and skilled to provide postnatal care for all newborns?   - Community health workers: - Auxiliary nurse midwives: - Nurses / auxiliary nurses: - Midwives / auxiliary midwives - Other cadre (please specify all): ………. |
| 3.3 What cadre of health care workers are authorized to prescribe and/or administer chlorhexidine for cord care at the community level?   - Community health workers: ☐Administer only ☐Prescribe and administer ☐ Prescribe only - Midwives ☐Administer only ☐Prescribe and administer ☐ Prescribe only - Auxiliary midwives: ☐Administer only ☐Prescribe and administer ☐ Prescribe only - Nurses: ☐Administer only ☐Prescribe and administer ☐ Prescribe only - Specialists (OB-G): ☐Administer only ☐Prescribe and administer ☐ Prescribe only - Physicians/clinicians ☐Administer only ☐Prescribe and administer ☐ Prescribe only - Other cadre (please specify all): ……… ☐Administer only ☐Prescribe and administer ☐ Prescribe only |
| 3. 4 Explain whether there are job descriptions and job aids for health workers at all levels of care, which reflect their role in assessing, caring for, and monitoring newborn babies at the health facility and at home. |
| 3.5 Are there competency-based training programmes through which the respective cadre of health care workers acquire the necessary knowledge and skills to provide basic newborn care for all newborns including recognition of danger signs at the facility and community levels?   - Community health workers: ☐Pre-service training ☐ In-service training - Nurses ☐Pre-service training ☐ In-service training - Midwives ☐Pre-service training ☐ In-service training - Auxiliary midwives: ☐Pre-service training ☐ In-service training - Clinical officers ☐Pre-service training ☐ In-service training - Physicians/clinicians ☐Pre-service training ☐ In-service training - Specialists (OB-G) ☐Pre-service training ☐ In-service training - Other cadre: …………. ☐Pre-service training ☐ In-service training |
| **Please provide a summary of key bottlenecks.** |
| **After responding to the questions above, please make an overall assessment of whether health workforce mechanisms in place for basic newborn care for all newborns is:**   - **Good** *(not a bottleneck to scale up)* - **Needs some improvements** *(minor bottleneck to scale up)* - **Needs major improvements** *(significant bottleneck to scale up)* - **Inadequate** *(very major bottleneck to scale up)* |
| **4. Essential medical products and technologies** |
| 4.1 Is chlorhexidine included in the National Essential Medicines List (NEML) for the indication of prevention of umbilical cord infections at the community level? If not, please explain why. |
| 4.2 Explain whether there are sufficient medical products (branded or generic) that can be used for the prevention of umbilical cord infections at the community level and explain if they are licensed. Please list the products. |
| 4.3. Explain if there are functional national or local systems in place to forecast accurately and distribute chlorhexidine according to the need for prevention of umbilical cord infections at the community level. |
| 4.4 Describe and explain the reasons for stock-outs of chlorhexidine at national and sub-national level in the last twelve months, if any. |
| **Please, provide a summary of key bottlenecks.** |
| **After responding to the questions above, please make an overall assessment of whether procurement and management systems in place for basic newborn care for all newborns is:**   - **Good** *(not a bottleneck to scale up)* - **Needs some improvements** *(minor bottleneck to scale up)* - **Needs major improvements** *(significant bottleneck to scale up)* - **Inadequate** *(very major bottleneck to scale up)* |
| **5. Health service delivery** |
| 5.1 Explain if the organization of newborn care services within health facilities allows for basic care of newborns in the first 24 hours after child birth and during postnatal care.   - Explain if there are a limited number of health facilities providing BNC for all newborns and what the balance is between urban and rural/remote areas. - Explain whether the enabling environment is adequate in delivering BNC (e.g. roads networks, hygiene standards, water and sanitation system in health facilities, etc.). - Describe whether there is a long waiting time for postnatal care visits in the health facilities and if the service available on a daily basis. - Explain if there is a public-private partnership that enhances delivery of newborn health care services. |
| 5.2 Explain if there are systems in place to promote the adherence to national standard and clinical protocols on basic newborn care (see section 1.2 on leadership and governance for relevant guidelines). For example:   - Explain if there are quality improvement mechanisms in place with standardized tools such as checklists for quality of basic newborn care for all newborns. - Describe specific efforts made by public and private (for-profit) health care facilities to promote and ensure quality basic newborn care. - Explain whether districts or health facilities conduct periodic reviews to ensure the provision of quality basic care for all newborns. |
| 5.3 Explain if there is a seamless continuum of postnatal care for mothers and newborns between the health facility and the home with at least 2 additional contacts, on day 3 and between day 7 and 14.  Explain if there is a system in place to train, supervise, and mentor community health workers providing newborn care services within 24 hours after birth and during postnatal visits in the communities.  Explain if the link between health facilities and the communities is strengthened by a functional referral system. If not, please explain. |
| 5.4 Describe other barriers to the delivery of basic newborn health care services that need to be addressed.  For example:   - Lack of information on clients’ needs to improve performance - Acceptability by health care workers about the use of chlorhexidine by mothers for cord care at the community level |
| **Please provide a summary of key bottlenecks.** |
| **After responding to the questions above, please make an overall assessment of whether health service delivery mechanisms in place to ensure for basic newborn care for all newborns is:**   - **Good** *(not a bottleneck to scale up)* - **Needs some improvements** *(minor bottleneck to scale up)* - **Needs major improvements** *(significant bottleneck to scale up)* - **Inadequate** *(very major bottleneck to scale up)* |
| **6. Health information systems** |
| 6.1 Describe the information available on basic newborn care coverage for all newborns. For example:   - Describe and list the indicators used to assess the coverage of basic newborn care (e.g. breastfeeding within 1 hour, number and timing of postnatal visits for newborns, etc.). Please specify the reference document including publication dates and page numbers. - Describe and specify any information on cord care including the use of chlorhexidine for cord care at the community level included in the HMIS. If not, please explain. |
| 6.2 Explain whether findings during postnatal care visits are included in standard clinical records or checklists. For example, explain if the use of chlorhexidine for cord care at the community level is included in standard clinical records or checklists such as birth records and safe childbirth checklists. |
| 6.3 Explain if the critical review of the appropriate basic newborn care for all newborns is included in protocols for clinical audits and perinatal death reviews. |
| **Please provide a summary of key bottlenecks.** |
| **After responding to the questions above, please make an overall assessment of whether the health information systems in place for basic newborn care for all newborns is:**   - **Good** *(not a bottleneck to scale up)* - **Needs some improvements** *(minor bottleneck to scale up)* - **Needs major improvements** *(significant bottleneck to scale up)* - **Inadequate** *(very major bottleneck to scale up)* |
| **7. Community ownership and participation** |
| 7.1 Explain if all newborns in need of basic care can use the services at all levels of care. For example:   - Explain whether postnatal visits are limited by socio-cultural barriers (misconceptions, beliefs, seclusion of newborns), lack of male involvement or long distance to health facilities. - Describe strategies that are in place to facilitate the use of newborn services by newborns living in rural and remote areas. - Describe how the referral mechanisms between the community and the health facilities are organized to facilitate timely referral and access to care for all newborns. |
| 7.2 Describe specific efforts in place to increase the awareness of the general public, adolescent girls, pregnant women and young couples of the benefits of timely recognition of a newborn with signs of illness and care seeking in a health facility. For example:   - Is information on the benefits of timely recognition of signs of illness and referral to an appropriate provider available in appropriate local language? - Do women and the general public know about that information? - Are there IEC materials available in appropriate local language? - What is the level of male involvement to facilitate and increase care seeking for newborns? |
| - 1. Describe other challenges faced by women that limit the use of quality basic newborn care services? - Is the use of chlorhexidine for cord care acceptable by women in the communities? - Is newborn mortality in health facilities a deterrent for families to seek care or accept referral? |
| **Please provide a summary of key bottlenecks.** |
| **After responding to the questions above, please make an overall assessment of whether community ownership and participation to enhance basic newborn care for all newborns is:**   - **Good** *(not a bottleneck to scale up)* - **Needs some improvements** *(minor bottleneck to scale up)* - **Needs major improvements** *(significant bottleneck to scale up)* - **Inadequate** *(very major bottleneck to scale up)* |

**IDENTIFICATION OF SOLUTIONS** **TO ADDRESS THE CHALLENGES**

*Please add sheets as appropriate*

| **Intervention 5: *BASIC NEWBORN CARE FOR ALL NEWBORNS - Focus on cleanliness/cord care, warmth, and feeding support*** | | |
| --- | --- | --- |
| **Summary of key bottlenecks *by order of priority*** | | Strategies and solutions to address identified challenges and bottlenecks |
| *Building block* | *Priority bottlenecks* |  |
| *Leadership and Governance* |  |  |
| *Health Finance* |  |  |
| *Health Workforce* |  |  |
| *Essential Medical products and Technologies* |  |  |
| *Health Service Delivery* |  |  |
| *Health Information Systems* |  |  |
| *Community Ownership and Participation* |  |  |

**Intervention 6: *NEONATAL RESUSCITATION***

***Focus on cleanliness/cord care, warmth, and feeding support***

Neonatal resuscitation is defined as the set of interventions at the time of birth to support the establishment of breathing and circulation. Basic resuscitation is done with a bag and mask.

| **1. Leadership and governance** |
| --- |
| 1.1Explain if the RMNCH plan/strategy includes specific actions for scaling-up neonatal resuscitation to save newborns. Please specify. If none exist, please explain why. |
| 1.2 Explain if national RMNCH policies are in place to ensure availability of neonatal resuscitation services in the country and if policies clearly indicate the need to integrate the services into EmOC services. If not, please explain. |
| 1.3 Describe the national standard treatment guidelines or clinical protocols on neonatal resuscitation by level of care (specify name of the document and year of publication). If none exist, please explain why.   - Explain if there are guidelines on immediate actions and actions after the baby is stable and breathing. - Explain if they specify key actions for the use of bag and mask supply and warmth. - Explain if there are guidelines for management of small/LBW babies and those who need prolonged assistance for breathing, including referral, if needed. - Explain if all recommendations are regularly updated and in line with current best practices (e.g. latest WHO guidelines). |
| 1.4 At what level of care is neonatal resuscitation recommended? Please specify the reference document.   - First level /outreach - Referral level (secondary and tertiary)   Describe the current recommendations for the community level in the country. |
| 1.5 Explain whether all relevant policies or regulations are aligned with the recommendations for neonatal resuscitation. For example, human resources policies. |
| **Please provide a summary of key bottlenecks.** |
| **After responding to the questions above, please make an overall assessment of whether leadership and governance mechanisms in place for neonatal resuscitation** **are:**   - **Good** *(not a bottleneck to scale up)* - **Need some improvements** *(minor bottleneck to scale up)* - **Need major improvements** *(significant bottleneck to scale up)* - **Inadequate** *(very major bottleneck to scale up)* |
| **2. Health financing** |
| - 1. Describe specific financing issues related to the delivery of neonatal resuscitation services at all recommended levels of care to all newborns, for example:   Explain if funds are sufficient to maintain emergency neonatal services and if they are sufficient to procure and distribute neonatal resuscitation devices such as bag and mask continuously in all health facilities at all recommended levels of care for all neonates in need.   - At the national level: - At the district or facility level: |
| - 1. Describe specific financing issues related to follow-up care after resuscitation, e.g. for unstable babies who need referral and further support. For example: - Describe financial barriers to referral to the appropriate level of care and/or hospital admission of the newborn. - Describe whether funds for the provision of transport for unstable newborns after resuscitation. |
| 2.3 Describe any financial barriers that prevent women from using any health facility that is able to perform neonatal resuscitation. For example:   - Describe out-of-pocket payments that are a barrier to care-seeking for women. - Describe user fees that represent a barrier to admission of women and their newborns |
| 2.4 Please explain any additional financial barriers to implement neonatal resuscitation services. For example: lack of public funds to re-organize some health facilities or lack of recovery costs. |
| **Please provide a summary of key bottlenecks.** |
| **After responding to the questions above, please make an overall assessment of whether the health financing mechanism in place for neonatal resuscitation of all newborns is:**   - **Good *(not a bottleneck to scale up)*** - **Needs some improvements *(minor bottleneck to scale up)*** - **Needs major improvements *(significant bottleneck to scale up)*** - **Inadequate *(very major bottleneck to scale up)*** |
| **3. Health workforce** |
| 3.1 Explain if there are sufficient numbers of competent health care workers who can perform neonatal resuscitation, at each level of care, where this intervention should be implemented. Issues may include:   - There are insufficient numbers of skilled health workers that are able to perform neonatal resuscitation in health facilities. - There might be sufficient numbers of trained health care workers, but they do not have the necessary competencies to perform neonatal resuscitation. - How is the distribution of personnel trained to provide neonatal resuscitation between rural and urban populations? Is there a plan for placement of professionals in underserved areas? |
| 3.2 What cadre of health care workers are trained/authorized to perform neonatal resuscitation in health facilities?   - Midwives - Auxiliary midwives - Nurses - Auxiliary nurses: - Physicians/clinicians - Specialists (OB-G) - Other cadre (please specify all): ………. |
| 3.3 Explain whether there are job descriptions and job aids for health workers at all levels of care, which reflect their role in performing neonatal resuscitation in the facilities. Explain the availability and accessibility of manuals detailing standards of practice for staff. |
| 3.4 Are there competency-based training programmes through which the respective cadre of health care workers acquire the necessary knowledge and skills to perform neonatal resuscitation?   - Nurses ☐Pre-service training ☐ In-service training - Midwives ☐Pre-service training ☐ In-service training - Auxiliary midwives: ☐Pre-service training ☐ In-service training - Clinical officers ☐Pre-service training ☐ In-service training - Physicians/clinicians ☐Pre-service training ☐ In-service training - Other cadre: …………. ☐Pre-service training ☐ In-service training   Explain if the contents of the training manuals are adapted according to international guidelines. Please specify the year of last update of training materials. |
| 3.5 What supervision and mentoring mechanisms are in place to ensure that trained health workers adequately perform neonatal resuscitation using bag and mask as per national guidelines? |
| **Please provide a summary of key bottlenecks.** |
| **After responding to the questions above, please make an overall assessment of whether health work force to perform neonatal resuscitation in the facilities is:**   - **Good** *(not a bottleneck to scale up)* - **Needs some improvements** *(minor bottleneck to scale up)* - **Needs major improvements** *(significant bottleneck to scale up)* - **Inadequate** *(very major bottleneck to scale up)* |
| **4. Essential medical products and technologies** |
| 4.1 Indicate whether ambu bags and masks are included in the National Essential Device List (NEDL) for the indication of neonatal resuscitation? |
| 4.2 Who are the main manufacturers of these devices? Explain whether sufficient medical products (branded or generic) that can be used for neonatal resuscitation are licensed. |
| 4.3 Explain and describe if there are functional national or local systems in place to accurately forecast and distribute these devices when needed. If not, please explain why. |
| 4.4 Describe and specify the causes of stock-outs of bag and masks for neonatal resuscitation at national and sub-national level in the last twelve months, if any.  Explain if there is a functional logistics information system able to detect the need of bags and masks in health facilities in need of the device. |
| 4.5 Provide additional challenges related to the procurement and distribution of bag and masks in health facilities. |
| **Please provide a summary of key bottlenecks.** |
| **After responding to the questions above, please make an overall assessment of whether the procurement and supply management system for neonatal resuscitation devices in health facilities is:**   - **Good** *(not a bottleneck to scale up)* - **Needs some improvements** *(minor bottleneck to scale up)* - **Needs major improvements** *(significant bottleneck to scale up)* - **Inadequate** *(very major bottleneck to scale up)* |
| **5. Health service delivery** |
| 5.1 Explain if neonatal resuscitation is available in facilities at all recommended levels of care. For example:   - Describe what types of health facilities perform more neonatal resuscitation and if it is performed in rural and remote areas. Explain if an inspection is performed regularly to ensure that bags and masks needed for neonatal resuscitation are present. - Explain if a management system is in place to ensure that neonatal resuscitation is available 24 hours a day. If not, please explain why. |
| 5.2 Describe the systems in place to promote the adherence to national standard and clinical protocols on neonatal resuscitation. For example:   - Explain if there are quality improvement mechanisms in place with standardized tools such as checklists to assess and enhance the quality of neonatal resuscitation services provided to newborns. - Explain whether assisted neonatal resuscitation is performed regularly and assessed every three to six months in health facilities and if there are manuals detailing standards of practice available and accessible for staff use. - Are perinatal death reviews conducted? - Are the standards maintained in public and private facilities to ensure quality care for all newborns in line with national guidelines? Please explain. - Describe the hygiene and infection prevention measures in place. |
| 5.3 Explain if there is a functional referral system to strengthen linkages between health facilities and the community. If not, why? Are communication systems in place between hospitals and ambulances? Explain whether ambulances services are functional and effective and if communication systems (radios/phones) are available for referrals? |
| 5.4 Describe other barriers to the performance of *neonatal resuscitation* in health facilities. |
| **Please provide a summary of key bottlenecks.** |
| **After responding to the questions above, please make an overall assessment of whether health service delivery for neonatal resuscitation is:**   - **Good** *(not a bottleneck to scale up)* - **Needs some improvements** *(minor bottleneck to scale up)* - **Needs major improvements** *(significant bottleneck to scale up)* - **Inadequate** *(very major bottleneck to scale up)* |
| **6. Health information systems** |
| 6.1 Explain if information is available on neonatal resuscitation in health facilities and what indicators are used by the routine system to monitor neonatal resuscitation. For example:   - Describe the indicators used to assess neonatal resuscitation and explain if any information is available on the number of neonatal resuscitations performed monthly or as a proportion of live births in health facilities. - Describe and specify any information on the use of bag and masks for neonatal resuscitation in the HMIS. |
| - 1. Explain whether the critical review of the quality of neonatal resuscitation services is included in protocols for perinatal death reviews. |
| - 1. Describe the completeness and quality of facility records on neonatal resuscitation activities. |
| **Please provide a summary of key bottlenecks.** |
| **After responding to the questions above, please make an overall assessment of whether monitoring and evaluation of neonatal services is:**   - **Good** *(not a bottleneck to scale up)* - **Needs some improvements** *(minor bottleneck to scale up)* - **Needs major improvements** *(significant bottleneck to scale up)* - **Inadequate** *(very major bottleneck to scale up)* |
| **7. Community ownership and partnership** |
| 7.1Describe any specific efforts to increase the awareness of the generic public, adolescent girls, pregnant women and young couples of the benefits of timely utilization of emergency newborn services in health facility:   - Are there IEC materials available in appropriate local language? - Describe male involvement in MNH issues and support in the use of maternal and newborn health services. |
| - 1. Describe other challenges faced by women that limited the use of neonatal resuscitation services. - Is emergency neonatal care affordable to poorer families? - Are adequate transportation options to medical care facilities available? - How much do knowledge women have on this topic? Do they know their rights? - Are gender issues or socio-cultural factors barriers to use of services? |
| 7.3 Describe the level of engagement of the communities to increase the use of emergency neonatal services during childbirth when needed. For example:   - Explain if community perspectives on health care have been taken into account and if community representatives are involved in perinatal audit committees and reviews. - Describe ways men/community leaders/women groups could be helpful in mobilizing and educating the community in care seeking |
| **Please provide a summary of key bottlenecks.** |
| **After responding to the questions above, please make an overall assessment of whether community ownership and partnership to enhance the use of neonatal resuscitation services is:**   - **Good** *(not a bottleneck to scale up)* - **Needs some improvements** *(minor bottleneck to scale up)* - **Needs major improvements** *(significant bottleneck to scale up)* - **Inadequate** *(very major bottleneck to scale up)* |

**IDENTIFICATION OF SOLUTIONS** **TO ADDRESS THE CHALLENGES**

*Please add sheets as appropriate*

| **Intervention 6: *NEONATAL RESUSCITATION*** | | |
| --- | --- | --- |
| **Summary of key bottlenecks *by order of priority*** | | Strategies and solutions to address identified challenges and bottlenecks |
| *Building block* | *Priority bottlenecks* |  |
| *Leadership and Governance* |  |  |
| *Health Finance* |  |  |
| *Health Workforce* |  |  |
| *Essential Medical products and Technologies* |  |  |
| *Health Service Delivery* |  |  |
| *Health Information Systems* |  |  |
| *Community Ownership and Participation* |  |  |

**Intervention 7: *KANGAROO MOTHER CARE***

***Focus on skin-to-skin, breastfeeding and feeding support for premature and small babies***

Kangaroo mother care (KMC) includes a number of interventions including continuous skin-to-skin contact in kangaroo position, support for frequent and exclusive breastfeeding or breast milk feeding. KMC is an effective intervention for clinically stable neonates weighing < 2000 g.

| **1. Leadership and governance** |
| --- |
| - 1. Explain if KMC been identified as a high impact intervention in the national RMNCH plan/strategy (specify the name of the documents),   Explain if the plan/strategy includes specific actions to introduce KMC in maternal and newborn health services.  Explain if there is a policy to introduce/implement KMC at scale. If not, please explain why. |
| 1.2 Explain if there are national standard treatment guidelines or clinical protocols covering KMC including support for feeding of low-birth-weight babies (Please specify name of the guidelines/clinical protocol document and year of publication), If none exist, please explain why. Do the guidelines:   - Specify how, when and where KMC should be implemented? - Contain a recommendation of how to organize health services to provide KMC? - Include information on feeding of low-birth weight babies? - Include milk banking? - In line with most recent WHO guidelines on KMC? |
| 1.3 If the use of KMC for stable low birth weight babies is recommended, please specify the level of care: [   - Primary level - First referral (secondary) level - Second referral (tertiary level) - Facility level initiation of KMC with follow-up at home |
| 1.4 If KMC is recommended, explain if all relevant policies or regulations are aligned with these recommendations. For example:   - Explain whether regulatory bodies for midwifery and/or nursing personnel recognize KMC as a high impact intervention and if they set standards and competencies for adequate care. - Explain whether documents on health facility standards foresee space for KMC and/or milk bank facilities. |
| **Please provide a summary of key bottlenecks.** |
| **After responding to the questions above, please make an overall assessment of whether leadership and governance related to KMC is:**   - **Good** *(not a bottleneck to scale up)* - **Needs some improvements** *(minor bottleneck to scale up)* - **Needs major improvements** *(significant bottleneck to scale up)* - **Inadequate** *(very major bottleneck to scale up)* |
| **2. Health financing** |
| 2.1 Describe specific health care financing issues related to the provision of KMC including the feeding of LBW babies:   - - Explain if there is an investment plan for scaling-up KMC as part of the RMNCH strategy   - Explain whether funding is available for health services to institutionalize KMC (e.g. trained personnel, food and lodging of mothers, and appropriate spaces and minimal supplies). |
| 2.2 Explain if there are sufficient funds available to establish and run milk banks in facilities to support KMC implementation. |
| 2.3 Describe any financial barriers for the women who are eligible to practice KMC, to benefit from this intervention. For example:   - Explain if it is feasible for mothers of LBW babies to agree to a prolonged stay in institutionalized care. - Explain whether food and lodging is available for mothers who are eligible for prolonged stay. |
| 2.4 Describe other financial barriers to scaling up KMC |
| **Please provide a summary of key bottlenecks.** |
| **After responding to the questions above, please make an overall assessment of whether health financing mechanisms for KMC are:**   - **Good** *(not a bottleneck to scale up)* - **Need some improvements** *(minor bottleneck to scale up)* - **Need major improvements** *(significant bottleneck to scale up)* - **Inadequate** *(very major bottleneck to scale up)* |
| **3. Health workforce** |
| 3.1 Explain if there are sufficient numbers of competent health care workers who can support KMC, at each level of care, where this intervention should be implemented. Issues may include:   - There is not sufficient midwifery or nursing personnel, who can support KMC including support for feeding of low birth weight babies - There might be sufficient numbers of knowledgeable, skilled and motivated health care workers, but they are not authorized to provide KMC - There might be sufficient numbers of trained health care workers, but they do not have the necessary competencies for supporting KMC including support for feeding of low birth weight babies - Insufficient competent staff to run milk banks |
| 3. 2 List which cadre of health care workers are authorized to support KMC including support for feeding low birth weight babies |
| 3. 3 Explain whether there are (standard) job descriptions for health personnel for all levels of care, which reflect their role in providing KMC. |
| 3.4 Are there competency-based training programmes through which the respective cadres of health care workers acquire the necessary knowledge and skills to support KMC?     - Auxiliary nurse midwives: ☐Pre-service training ☐ In-service training - Nurses: ☐Pre-service training ☐ In-service training - Midwives: ☐Pre-service training ☐ In-service training - Associate clinicians: ☐Pre-service training ☐ In-service training - Adv.-level associate clinicians: ☐Pre-service training ☐ In-service training - Non-specialist doctors: ☐Pre-service training ☐ In-service training - Other cadre: …………. ☐Pre-service training ☐ In-service training |
| 3.5 Explain if there are supervision and/or mentoring guidelines and systems in place to support the provision of effective KMC including support for feeding of LBW babies. |
| **Please provide a summary of key bottlenecks.** |
| **After responding to the questions above, please make an overall assessment of whether the health workforce for implementation of KMC is:**   - **Good** *(not a bottleneck to scale up)* - **Needs some improvements** *(minor bottleneck to scale up)* - **Needs major improvements** *(significant bottleneck to scale up)* - **Inadequate** *(very major bottleneck to scale up)* |
| **4. Essential medical products and technologies** |
| 4.1 Are the following basic supplies available for supporting KMC and feeding of low birth weight babies?  ☐ Cups  ☐ Nasogastric tubes  ☐ Breast milk containers  ☐ Pasteurizers for breastmilk  ☐ Refrigerators for breast milk storage |
| **Please provide a summary of the key bottlenecks related to procurement and supply management of basic supplies.** |
| **After responding to the questions above, please make an overall assessment of whether procurement and supply of essential supplies for KMC is:**   - **Good** *(not a bottleneck to scale up)* - **Needs some improvements** *(minor bottleneck to scale up)* - **Needs major improvements** *(significant bottleneck to scale up)* - **Inadequate** *(very major bottleneck to scale up)* |
| **5. Health service delivery** |
| 5.1 Describe the systems in place to promote the adherence to national standard treatment guidelines or clinical protocols on the use of kangaroo mother care. If none exists please explain why. For example:   - Describe the quality improvement programmes aiming at increasing implementation of KMC. - Describe specific efforts taken to promote and monitor KMC in both public and private (for-profit) health care facilities. |
| 5.2 What is the lowest level of care where KMC, including support for feeding of LBW babies, is implemented adequately? For example:   - Is it available at primary level? - Do multiple referrals of LBW babies lead to unnecessary delays? - Is there a mechanism to support and follow-up women and babies for KMC in the community? |
| 5.3 Explain whether the organization of health care within facilities prevents women from practicing KMC. For example:   - Describe whether health facilities providing maternal and newborn care are able to create adequate spaces for supporting and monitoring mothers and babies who are eligible for KMC. - Describe logistic constraints to lodging mothers and babies for KMC in health facilities. |
| 5.4 Explain whether appropriate information is provided by health care providers to mothers to increase the acceptance of KMC as a life-saving intervention. For example:   - Information on the acceptability of KMC as an effective intervention by health workers - Organization of activities for women and their communities by health care workers to increase knowledge on KMC issues |
| **Please provide a summary of key bottlenecks.** |
| **After responding to the questions above, please make an overall assessment of whether the health service delivery for KMC is:**   - **Good** *(not a bottleneck to scale up)* - **Needs some improvements** *(minor bottleneck to scale up)* - **Needs major improvements** *(significant bottleneck to scale up)* - **Inadequate** *(very major bottleneck to scale up)* |
| **6. Health information systems** |
| 6.1 Indicate whether information is available on the coverage of KMC For example:   - What are the indicators used to assess the coverage of KMC? Please specify the reference document for the indicators used, including publication dates and pages. - Specify any information on KMC included in the HMIS. |
| 6.2 Explain whether KMC is included in standard clinical records or checklists (e.g. birth records or child health card). Please specify the document. |
| **Please provide a summary of key bottlenecks.** |
| **After responding to the questions above, please make an overall assessment of whether the health information system for KMC is:**   - **Good** *(not a bottleneck to scale up)* - **Needs some improvements** *(minor bottleneck to scale up)* - **Needs major improvements** *(significant bottleneck to scale up)* - **Inadequate** *(very major bottleneck to scale up)* |
| **7. Community ownership and participation** |
| 7.1 Explain whether all newborns in need of basic care can use KMC services at all levels of care. For example:   - Describe whether the use of KMC is limited by socio-cultural barriers (misconceptions, beliefs, seclusion of newborns), lack of male involvement or long distances to health facilities. - Explain whether postnatal visits are conducted to ensure the continuity of KMC from the facility to the community. - Describes strategies in place to facilitate the use of KMC by newborns living in rural and remote areas. - Describe how the referral mechanisms between the community and the health facilities are organized to facilitate timely referral and access to care by all newborns. |
| 7.2 Describe specific efforts to increase the awareness of the general public, adolescent girls, pregnant women and young couples of the benefits of KMC. For example:   - Is information on the benefits of KMC by an appropriate provider available in appropriate local language? Are there IEC materials available in appropriate local language? - Do women and the general public know about that information? - Describe the level of male involvement to facilitate implementation of KMC |
| 7.3 Describe other barriers to the use of KMC that need to be addressed. For example:   - Do attitudes of health care workers that affect the pickup of this intervention? Is cultural perception an issue? Please explain. |
| **Please provide a summary of key bottlenecks.** |
| **After responding to the questions above, please make an overall assessment of whether community ownership and participation to enhance KMC is:**   - **Good** *(not a bottleneck to scale up)* - **Needs some improvements** *(minor bottleneck to scale up)* - **Needs major improvements** *(significant bottleneck to scale up)* - **Inadequate** *(very major bottleneck to scale up)* |

**IDENTIFICATION OF SOLUTIONS** **TO ADDRESS THE CHALLENGES**

*Please add sheets as appropriate*

| **Intervention 7: *KANGAROO MOTHER CARE - Focus on skin-to-skin, breastfeeding and speeding support for premature and small babies*** | | |
| --- | --- | --- |
| **Summary of key bottlenecks *by order of priority*** | | Strategies and solutions to address identified challenges and bottlenecks |
| *Building block* | *Priority bottlenecks* |  |
| *Leadership and Governance* |  |  |
| *Health Finance* |  |  |
| *Health Workforce* |  |  |
| *Essential Medical products and Technologies* |  |  |
| *Health Service Delivery* |  |  |
| *Health Information Systems* |  |  |
| *Community Ownership and Participation* |  |  |

**Intervention 8: *MANAGEMENT OF SEVERE NEWBORN INFECTIONS***

***Focus on the use of injectable antibiotics***

The management of severe infections in the newborn requires proper assessment and the use of injectable antibiotics as well as full supportive care – this analysis looks at the first two aspects. The latter will be analysed as a separate intervention package.

| **1. Leadership and governance** |
| --- |
| 1.1 Explain if severe infections have been identified as major cause of preventable newborn deaths in the national RMNCH plan/strategy (specify the name of the documents), and if the plan/strategy includes specific actions to avert those deaths through identification and treatment of newborn infections including the use of injectable antibiotics. If not, please explain. |
| 1.2 Explain if there are national standard treatment guidelines or clinical protocols covering identification and management of neonatal infections (specify name of the guidelines/clinical protocol document and year of publication).   - Explain if they include recommendations of minimum stay period of stay before discharge for facility births during which signs of infections are monitored. - Explain if there are recommendations on scheduled postnatal visits, including home visits in the first week of life, during which newborns are assessed for signs of infection. - Explain if they recommend a clinical algorithm for the assessment of the newborn and the identification of severe newborn infection - Do they recommend injectable antibiotics for treatment of severe newborn infections? - Do they include the recommendation to refer severe newborn infections to hospital for full supportive care? - Are all recommendations in line with current best practices (e.g. latest WHO guidelines)? - Are they regularly updated? |
| 1.3 At what level of care is the assessment of the newborn with signs of illness recommended?   - Community level? - First level/ outreach? - Referral? |
| 1.4 At what level of care is the management and treatment of severe newborn infections with of injectable antibiotics recommended?   - Community level? - First level/ outreach? - Referral? |
| 1.5 Explain whether all relevant policies or regulations are aligned with the recommendations for identification and treatment of severe newborn infections. For example:   - Explain if health facility discharge policies are in line with national guidelines. - Describe which cadres of health personnel are authorized and trained to manage severe newborn infection. - Explain whether health workers who assess and manage newborn babies are authorized to prescribe and administer injectable antibiotics for severe newborn infections (more details in section on human resources) - Explain whether hospital (public and private) policies are updated to reflect national guidelines |
| **Please provide a summary of key bottlenecks.** |
| **After responding to the questions above, please make an overall assessment of whether leadership and governance for the identification and management of severe newborn infection is:**   - **Good** *(not a bottleneck to scale up)* - **Needs some improvements** *(minor bottleneck to scale up)* - **Needs major improvements** *(significant bottleneck to scale up)* - **Inadequate** *(very major bottleneck to scale up)* |
| **2. Health financing** |
| 2.1 Describe specific health care financing issues related to the procurement and/or distribution of injectable antibiotics for the intended level of care. For example:   - *At the national level:*   - Explain whether funds are sufficient in centralized procurement and distribution systems to procure and distribute injectable antibiotics in a continuous and sustainable matter to all health facilities where they are required. - *At the district or facility level:*   - Describe whether the availability of injectable antibiotics poses a problem.   - Explain whether funds are sufficient in decentralized procurement and distribution systems to procure and distribute injectable antibiotics in a continuous and sustainable matter to all health facilities where they are required. |
| - 1. Explain if there is sufficient funding to implement home visitation programmes to detect newborns with infections. |
| 2.3 Describe any financial barriers for mothers to seek care for their newborn babies, or for newborns to receive injectable antibiotics when they need them. For example:   - Describe financial barriers to routine postnatal care visits (e.g. transport costs and out-of-pocket payments). - Explain how financial access is a barrier to care-seeking for sick newborns (e.g. transport costs and out-of-pocket payments). - Describe financial access barriers to referral to hospital and hospital-based full supportive care (e.g. transport costs, out-of-pocket expenditures, loss of income, etc.) - Explain whether medicines and/or syringes need to be paid for in addition to general consultation or treatment fees. - Do patients/families need to purchase injectable antibiotics themselves, because they are not provided by the health care provider/facility? - Does the price of the recommended drug represent a financial barrier? - Describe any additional fees associated with this intervention (e.g. the need to pay for a prescription or a specialist consultation fee). |
| 2.4 Describe other financial barriers to scaling up the management and treatment of severe newborn infections |
| **Please provide a summary of key bottlenecks.** |
| **After responding to the questions above, please make an overall assessment of whether health financing for the identification and management of severe newborn infection is:**   - **Good** *(not a bottleneck to scale up)* - **Needs some improvements** *(minor bottleneck to scale up)* - **Needs major improvements** *(significant bottleneck to scale up)* - **Inadequate** *(very major bottleneck to scale up)* |
| **3. Health workforce** |
| 3.1 Explain whether there are sufficient numbers of competent health care workers who can identify and manage severe newborn infections, at each level of care, where this intervention should be implemented. Issues may include:   - There is insufficient health personnel to provide postnatal care and recognize newborns with signs of severe infections - There might be sufficient numbers of motivated health care workers, but they do not have the necessary competencies for the assessment and recognition of the newborns with signs of severe infection - There might be sufficient numbers of knowledgeable, skilled and motivated health care workers, but they are not authorized to prescribe or administer injectable antibiotics - How is the distribution of skilled personnel between rural and urban populations? |
| 3.2 What cadre of health care workers are authorized to treat severe newborn infections and prescribe and/or administer injectable antibiotics?   - Midwives ☐Administer only ☐Prescribe and administer ☐ Prescribe only - Nurses: ☐Administer only ☐Prescribe and administer ☐ Prescribe only - Auxiliary midwives: ☐Administer only ☐Prescribe and administer ☐ Prescribe only - Auxiliary nurses: ☐Administer only ☐Prescribe and administer ☐ Prescribe only - Physicians/clinicians ☐Administer only ☐Prescribe and administer ☐ Prescribe only - Other cadre (please specify all): ……… ☐Administer only ☐Prescribe and administer ☐ Prescribe only |
| 3. 3 Explain whether there are (standard) job descriptions for health care workers at all levels of care, which reflect the role in assessing newborn babies, managing severe newborn infections, and prescribing and /or administering injectable antibiotics. |
| 3.4 Are there competency-based training programmes through which the respective cadres of health care workers acquire the necessary knowledge and skills to provide postnatal care, identify newborns with signs of severe illness and prescribe and or administer injectable antibiotics?     - Nurses: ☐Pre-service training ☐ In-service training - Midwives: ☐Pre-service training ☐ In-service training - Specialist doctor: ☐Pre-service training ☐ In-service training - Non-specialist doctors: ☐Pre-service training ☐ In-service training - Other cadre: ☐Pre-service training ☐ In-service training |
| 3.5 Explain if there is supervision and/or mentoring guidelines and systems in place to ensure that quality of postnatal care and management of severe newborn infections is up to standard. |
| **Please provide a summary of key bottlenecks.** |
| **After responding to the questions above, please make an overall assessment of whether the health workforce for the identification and management of severe newborn infection is:**   - **Good** *(not a bottleneck to scale up)* - **Needs some improvements** *(minor bottleneck to scale up)* - **Needs major improvements** *(significant bottleneck to scale up)* - **Inadequate** *(very major bottleneck to scale up)* |
| **4. Essential medical products and technologies** |
| 4.1 Are the following injectable antibiotics included in the National Essential Medicines List (NEML) for the indication of management of severe newborn infections?  ☐ Ampicillin  ☐ Gentamycin  ☐Benzathine penicillin  ☐ Procaine penicillin  ☐Ceftriaxone  ☐ Other, please specify …… |
| 4.2 At what level of care is the use of injectable antibiotics recommended in the NEML? Please specify the level of care: e.g.:   - Primary level - First referral (secondary) Level - Second referral (tertiary level) |
| 4.3 Explain whether there are sufficient medical products (branded or generic) that can be used for treatment of severe newborn infection and if they licensed. Please specify products. |
| 4.4 Explain whether there are national or local systems in place to forecast accurately the need for injectable antibiotics for management of severe newborn infections. |
| 4.5 Describe and explain the reasons for stock-outs of injectable antibiotics at national and sub-national level in the last twelve months, if any. |
| **Please provide a summary of key bottlenecks.** |
| **After responding to the questions above, please make an overall assessment of whether the availability of injectable antibiotics for management of severe newborn infections is:**   - **Good** *(not a bottleneck to scale up)* - **Needs some improvements** *(minor bottleneck to scale up)* - **Needs major improvements** *(significant bottleneck to scale up)* - **Inadequate** *(very major bottleneck to scale up)* |
| **5. Health service delivery** |
| 5.1 Describe the systems in place to promote the adherence to national standard treatment guidelines or clinical protocols on identification and management of severe newborn infections (see section 1.2 on leadership and governance for relevant guidelines). If none exists, please explain why. For example:   - Describe quality improvement mechanisms that are in place for quality of newborn care including standardized tools such as checklists. - Describe specific efforts for public and private (for-profit) health care facilities. |
| 5.2 What is the lowest level of care where injectable antibiotics for management of severe neonatal infection are administered adequate? For example:   - Is it available at primary level? - Do multiple referrals lead to unnecessary delays? - Are timely referrals to the level of care where injectable antibiotics are given feasible? |
| 5.3 Explain whether the management of newborn sepsis/infections is integrated into integrated community case management of childhood illnesses guidelines. Please, specify the name of the document. |
| - 1. Describe the main issues faced by the health system to identify and treat neonatal infections in the community. |
| **Please provide a summary of key bottlenecks.** |
| **After responding to the questions above, please make an overall assessment of whether the health service delivery for the identification and management of severe newborn infections is:**   - **Good** *(not a bottleneck to scale up)* - **Needs some improvements** *(minor bottleneck to scale up)* - **Needs major improvements** *(significant bottleneck to scale up)* - **Inadequate** *(very major bottleneck to scale up)* |
| **6. Health information systems** |
| 6.1 Explain whether information is available on the coverage of postnatal care contacts and if information is available on the management of newborns with severe infection.   - What are the indicators used to assess the coverage of the intervention (use of injectable antibiotics)? Please specify the reference document for the indicators used, including publication dates and the page. - Specify any information included in the HMIS on postnatal contacts at the community level and facility level is recorded. - Specify any information included in the HMIS on the management of severe newborn infection |
| 6.2 Explain whether the findings of postnatal care contacts are included in standard clinical records or checklist.  Describe if use of injectable antibiotics for severe newborn infection is included in standard clinical records or checklists, e.g.: birth records, safe childbirth checklists. |
| - 1. Describe the critical review of the appropriate management of severe newborn infection included in protocols for clinical audits and perinatal death reviews |
| **Please provide a summary of key bottlenecks.** |
| **After responding to the questions above, please make an overall assessment of whether the health information system on the management of severe newborn infections is:**   - **Good** *(not a bottleneck to scale up)* - **Needs some improvements** *(minor bottleneck to scale up)* - **Needs major improvements** *(significant bottleneck to scale up)* - **Inadequate** *(very major bottleneck to scale up)* |
| **7. Community ownership and participation** |
| 7.1 Explain whether all newborns seeking care during the postnatal period for management of severe infection are able to access services at all levels of care For example:   - Explain whether socio-cultural barriers (misconceptions, beliefs, seclusion of newborns), lack of male involvement or long distance to health facilities limit use of services. - Describe strategies that are in place to facilitate the use of services by newborns living in rural and remote areas. - Explain how the referral mechanisms between the community and health facilities are organized to facilitate timely referral and access to care by all newborns. |
| 7.2Describe specific efforts to increase the awareness of the general public, adolescent girls, pregnant women and young couples on danger signs, the benefits of care seeking and postnatal visits. For example:   - Is information on the benefits of care seeking provided by an appropriate provider available in appropriate local language? Are there IEC materials available in appropriate local language? - Do women and the general public know about postnatal visits? - Describe the level of male involvement to facilitate care seeking for management and treatment of neonatal infections during the first week after birth. |
| 7.3 Describe other barriers to the use of postnatal care services for newborns that need to be addressed. For example:   - Describe health care workers’ attitudes that hinder the pickup of this intervention. - Describe any cultural perception issues. - Explain whether care seeking at the hospital during the first week after birth is acceptable in the community. |
| **Please provide a summary of key bottlenecks.** |
| **After responding to the questions above, please make an overall assessment of whether community ownership and participation to facilitate care seeking for management of severe newborn infections is:**   - **Good** *(not a bottleneck to scale up)* - **Needs some improvements** *(minor bottleneck to scale up)* - **Needs major improvements** *(significant bottleneck to scale up)* - **Inadequate** *(very major bottleneck to scale up)* |

**IDENTIFICATION OF SOLUTIONS** **TO ADDRESS THE CHALLENGES**

*Please add sheets as appropriate*

| **Intervention 8: *MANAGEMENT OF SEVERE NEWBORN INFECTIONS - Focus on the use of injectable antibiotics*** | | |
| --- | --- | --- |
| **Summary of key bottlenecks *by order of priority*** | | Strategies and solutions to address identified challenges and bottlenecks |
| *Building block* | *Priority bottlenecks* |  |
| *Leadership and Governance* |  |  |
| *Health Finance* |  |  |
| *Health Workforce* |  |  |
| *Essential Medical products and Technologies* |  |  |
| *Health Service Delivery* |  |  |
| *Health Information Systems* |  |  |
| *Community Ownership and Participation* |  |  |

**Intervention 9: *INPATIENT SUPPORTIVE CARE FOR SICK AND SMALL NEWBORNS***

***Focus on IV fluids, feeding support and safe oxygen***

Severely sick newborns with severe infections or who are too small to maintain their body temperature, to breath or to feed actively need full supportive in-patient care. This includes a number of interventions, including regular monitoring and assessments by health workers. As tracers for full supportive care, however, the following interventions have been identified: provision of IV fluids, intragastric tube feeding (IGTF), and safe oxygen administration.

| **1. Leadership and governance** |
| --- |
| - 1. Explain if full supportive in-patient care for severely sick newborns been identified as a priority intervention to avert preventable newborn deaths in the national RMNCH plan/strategy (specify the name of the documents).   If so, does the plan/strategy include specific actions for in-patient supportive care for severely sick and small newborns? |
| 1.2 Explain if there are national standard treatment guidelines or clinical protocols covering full supportive care for severely sick and small newborns after delivery. (Specify name of the guidelines/clinical protocol document and year of publication).   - Describe whether they address the most common potentially lethal conditions requiring in-patient care, i.e. severe infections (e.g. sepsis/meningitis) and complications of prematurity. - Describe whether they address the need for feeding support for severely sick newborns, including intragastric tube feeding? Do they promote family –centered care? - Explain whether all recommendations are regularly updated and in line with current best practices (e.g. latest WHO guidelines). |
| 1.3 At what level of care is inpatient care for sick and small/LBW babies recommended?   - First referral level (e.g. district hospital)? - Second referral level (e.g. with specialized care available)? - Tertiary level? |
| 1.4 Describe whether all relevant policies or regulations are aligned with the recommendations for inpatient care for sick and small/LBW babies. For example:   - Explain how/whether health facilities are organized and equipped for appropriate inpatient care and monitoring for sick and small/LBW babies in line with national guidelines. - Explain how health facilities maintain hygiene standards to ensure quality care for sick and small/LBW newborns. - Describe whether there is a cadre of health personnel authorized and trained to perform extra care for sick and small/LBW newborns. Explain whether health workers who assess and manage these newborns provide adequate extra support for feeding (including exclusive breastfeeding) and warmth? (More details in section on human resources). |
| **Please provide a summary of key bottlenecks.** |
| **After responding to the questions above, please make an overall assessment of whether leadership and governance mechanisms in place for inpatient care for sick and small/LBW newborns are:**   - **Good** *(not a bottleneck to scale up)* - **Need some improvements** *(minor bottleneck to scale up)* - **Need major improvements** *(significant bottleneck to scale up)* - **Inadequate** *(very major bottleneck to scale up)* |
| **2. Health financing** |
| 2.1 Describe any specific financing issues related to the implementation of inpatient care services for sick and small/LBW newborns at all recommended level of care. For example:   - *At the national level:*   - Explain whether there are sufficient funds in centralized procurement and distribution systems to procure and distribute necessary equipment (IV fluids and safe oxygen) at all recommended levels of care to continuously provide inpatient care to sick and small/LBW babies at all health facilities where they are required. - *At the district or facility level:*   - Explain whether there are sufficient funds in decentralized procurement and distribution systems, to procure and distribute necessary equipment (IV fluids and safe oxygen) at all recommended levels of care to continuously provide inpatient care to sick and small/LBW babies at all health facilities where they are required. |
| 2.2 Describe any financial barriers that prevent sick and small/LBW newborns from receiving appropriate care at the health facility. For example:   - Describe barriers to care-seeking for all sick and small/LBW newborns as a result of out-of-pocket payments. . - Explain whether user fees represent a barrier to admission of sick newborns and if the costs related to extra care for small/LBW babies are an issue for the clients who need it (e.g. fees for a neonatologist). - Do patients/families need to purchase drugs to benefit from this service? |
| 2.3 Describe other financial barriers to the expansion of newborn health services for inpatient care of sick and small/LBW newborns in all recommended health facilities. |
| **Please provide a summary of key bottlenecks.** |
| **After responding to the questions above, please make an overall assessment of whether health financing for inpatient care for sick and small/LBW newborns is:**   - **Good** *(not a bottleneck to scale up)* - **Needs some improvements** *(minor bottleneck to scale up)* - **Needs major improvements** *(significant bottleneck to scale up)* - **Inadequate** *(very major bottleneck to scale up)* |
| **3. Health workforce** |
| 3.1 Explain whether there are sufficient numbers of competent health care workers who can provide inpatient care for sick and small/LBW babies, at each level of care, where this intervention should be implemented. Issues may include:   - There are insufficient health workers to provide full supportive newborn care in the health facilities. - There might be sufficient numbers of trained health care workers, but they do not have the necessary competencies to provide full supportive care. - How is the distribution of skilled personnel trained to provide extra care for sick and small newborns between rural and urban populations? Is there an HR strategy to expand BNC to all newborns living in remote areas through community health workers? - Are there policies in place to insure monitoring and handling of sick/small babies by competent staff around the clock? |
| 3.2 What cadre of health care workers are authorized and skilled to provide extra newborn care to sick and small/LBW babies with: (please, check for each of these intervention)  ***IV fluids Oxygen IGT feeding***   - Nurses - Midwives - Auxiliary midwives - Physicians/Clinicians - Other cadre (please specify all): ………. |
| 3.3 What cadre of health care workers are authorized to prescribe and/or administer *IV fluids* to sick and small newborns?   - Midwives ☐Administer only ☐Prescribe and administer ☐ Prescribe only - Auxiliary midwives: ☐Administer only ☐Prescribe and administer ☐ Prescribe only - Nurses: ☐Administer only ☐Prescribe and administer ☐ Prescribe only - Physicians/clinicians ☐Administer only ☐Prescribe and administer ☐ Prescribe only - Other cadre (please specify all): ……… ☐Administer only ☐Prescribe and administer ☐ Prescribe only   What cadre of health care workers are authorized to prescribe and/or administer *oxygen* to sick and small newborns?   - Midwives ☐Administer only ☐Prescribe and administer ☐ Prescribe only - Auxiliary midwives: ☐Administer only ☐Prescribe and administer ☐ Prescribe only - Nurses: ☐Administer only ☐Prescribe and administer ☐ Prescribe only - Physicians/clinicians ☐Administer only ☐Prescribe and administer ☐ Prescribe only - Other cadre (please specify all): ……… ☐Administer only ☐Prescribe and administer ☐ Prescribe only   What cadre of health care workers are authorized to prescribe and/or administer *IGT feeding* to sick and small newborns?   - Midwives ☐Administer only ☐Prescribe and administer ☐ Prescribe only - Auxiliary midwives: ☐Administer only ☐Prescribe and administer ☐ Prescribe only - Nurses: ☐Administer only ☐Prescribe and administer ☐ Prescribe only - Physicians/clinicians ☐Administer only ☐Prescribe and administer ☐ Prescribe only - Other cadre (please specify all): ……… ☐Administer only ☐Prescribe and administer ☐ Prescribe only |
| 3. 4 Explain whether there are job descriptions and job aids for health workers at all levels of care, which reflect their role in assessing, caring for, and monitoring sick and small/LBW newborn babies at the health facility. |
| 3.5 Are there competency-based training programmes through which the respective cadre of health care workers acquire the necessary knowledge and skills to provide *IV fluids*?   - Nurses ☐Pre-service training ☐ In-service training - Midwives ☐Pre-service training ☐ In-service training - Auxiliary midwives ☐Pre-service training ☐ In-service training - Auxiliary nurses ☐Pre-service training ☐ In-service training - Clinical officers ☐Pre-service training ☐ In-service training - Physicians/clinicians ☐Pre-service training ☐ In-service training - Other cadre: …………. ☐Pre-service training ☐ In-service training   Are there competency-based training programmes through which the respective cadre of health care workers acquire the necessary knowledge and skills to provide *oxygen*?   - Nurses ☐Pre-service training ☐ In-service training - Midwives ☐Pre-service training ☐ In-service training - Auxiliary midwives ☐Pre-service training ☐ In-service training - Auxiliary nurses ☐Pre-service training ☐ In-service training - Clinical officers ☐Pre-service training ☐ In-service training - Physicians/clinicians ☐Pre-service training ☐ In-service training - Other cadre: …………. ☐Pre-service training ☐ In-service training   Are there competency-based training programmes through which the respective cadre of health care workers acquire the necessary knowledge and skills to provide *IGT feeding*?   - Nurses ☐Pre-service training ☐ In-service training - Midwives ☐Pre-service training ☐ In-service training - Auxiliary midwives ☐Pre-service training ☐ In-service training - Auxiliary nurses ☐Pre-service training ☐ In-service training - Clinical officers ☐Pre-service training ☐ In-service training - Physicians/clinicians ☐Pre-service training ☐ In-service training - Other cadre: …………. ☐Pre-service training ☐ In-service training |
| **Please provide a summary of key bottlenecks.** |
| **After responding to the questions above, please make an overall assessment of whether health workforce mechanisms in place for inpatient care for sick and small/LBW newborns are:**   - **Good** *(not a bottleneck to scale up)* - **Need some improvements** *(minor bottleneck to scale up)* - **Need major improvements** *(significant bottleneck to scale up)* - **Inadequate** *(very major bottleneck to scale up)***Good** |
| **4. Essential medical products and technologies** |
| 4.1 Are the following appropriate IV fluids included in the National Essential Medicines List (NEML) for the indication of management of sick and small newborns?  ☐ 10% Glucose  ☐ 0.45 NaCl/5% glucose  ☐ 0.18% NaCl/4% glucose |
| 4.2 Explain if oxygen is included in the National Essential Medicines List (NEML) for the indication of the management of sick and small newborns and if all necessary equipment for safe oxygen therapy is included in the national essential commodities list (i.e. oxygen concentrators, blenders, tubes and nasal prongs). |
| 4.3 Explain whether sufficient medical products (branded or generic), that can be used for treatment of sick and small/LBW newborns, are licensed. |
| 4.4 Explain if there are functional national or local systems in place to accurate forecasting and distribution of IV fluids and oxygen for the management of sick and small/LBW newborns in health facilities. |
| 4.5 Describe and explain the reasons for stock-outs of IV fluids and oxygen at national and sub-national levels in the last twelve months, if any. |
| **Please provide a summary of key bottlenecks.** |
| **After responding to the questions above, please make an overall assessment of whether procurement and management systems in place for inpatient care for sick and small/LBW newborns are:**   - **Good** *(not a bottleneck to scale up)* - **Need some improvements** *(minor bottleneck to scale up)* - **Need major improvements** *(significant bottleneck to scale up)* - **Inadequate** *(very major bottleneck to scale up)* |
| **5. Health service delivery** |
| 5.1 Explain whether the organization of newborn care services within health facilities allows for full supportive care of sick and small/LBW newborns.   - Are there a limited number of health facilities that provide extra care for sick and small/LBW babies? What is the balance between urban and rural/remote areas? - Is the service available on a daily basis? - Is there a clearly defined supportive environment in place for extra support for feeding methods, extra support for warmth to sick and small/LBW babies? - Is there a public-private partnership that enhances delivery of newborn health care services? |
| 5.2 Describe the systems in place to promote the adherence to national standard and clinical protocols on inpatient/extra care for sick and small/newborns (see section 1.2 on leadership and governance for relevant guidelines). For example:   - Describe quality improvement mechanisms in place with standardized tools such as check lists for quality of inpatient care for sick and small/LBW babies. - Explain specific efforts made by public and private (for-profit) health care facilities to promote and ensure quality basic newborn care. - Explain whether districts or health facilities conduct periodic reviews to ensure the provision of quality inpatient care for sick and small newborns. - Describe guidelines available and used by staff to improve the quality of inpatient care services for newborns. |
| 5.3 Describe other barriers to the delivery of basic newborn health care services that need to be addressed. For example:   - Lack of information on clients’ needs for improved performance - Health care workers’ attitudes - Does the country provide family-centered care for mothers and newborns? |
| **Please provide a summary of key bottlenecks.** |
| **After responding to the questions above, please make an overall assessment of whether health service delivery in place for full supportive care for sick and small/LBW newborns is:**   - **Good** *(not a bottleneck to scale up)* - **Needs some improvements** *(minor bottleneck to scale up)* - **Needs major improvements** *(significant bottleneck to scale up)* - **Inadequate** *(very major bottleneck to scale up)* |
| **6. Health information systems** |
| 6.1 Explain whether there is information available on extra newborn care coverage for sick and small newborns. For example:   - What are the indicators used to track the sick or small/LBW newborns that received extra care (number of newborns hospitalized?) or to record the weight of small/LBW babies assessed? Please specify the reference document for the indicators used, including publication dates and the page. |
| 6.2 Explain whether the findings observed during inpatient care for sick and small/LBW newborns are included in standard clinical records or checklists. |
| - 1. Explain whether the critical review of appropriate inpatient care for sick and small/LBW babies is included in protocols for clinical audits and perinatal death reviews. |
| **Please provide a summary of key bottlenecks.** |
| **After responding to the questions above, please make an overall assessment of whether health information systems in place to assess and monitor full supportive care for sick and small/LBW newborns are:**   - **Good** *(not a bottleneck to scale up)* - **Need some improvements** *(minor bottleneck to scale up)* - **Need major improvements** *(significant bottleneck to scale up)* - **Inadequate** *(very major bottleneck to scale up)* |
| **7. Community ownership and participation** |
| 7.1 Explain whether sick and small/LBW babies in need of extra neonatal care can use the services at all levels of care. For example:   - Explain whether care seeking is limited by socio-cultural barriers (misconceptions, beliefs, seclusion of newborns, etc.), lack of male involvement or long distance to health facilities. - Describe strategies in place to facilitate the use of inpatient neonatal care services by sick and small/LBW newborns living in rural and remote areas. - Describe the referral mechanisms in place between the community and health facilities organized to facilitate timely referral and access to care for all newborns. |
| 7.2 Describe specific efforts to increase the awareness of the general public, adolescent girls, pregnant women and young couples of the benefits of (1) timely recognition of a newborn with LBW or with signs of illness and (2) timely care seeking to a health facility. For example:   - Is information on the benefits of timely recognition of signs of illness and referral to an appropriate provider available in the appropriate local language? - Do women and the general public know about that information? - Are there IEC materials available in the appropriate local language? - What is the level of male involvement to facilitate care seeking for sick and small/LBW newborns? |
| - 1. Describe other challenges faced by women that limit the use of inpatient neonatal care services. |
| **Please provide a summary of key bottlenecks.** |
| **After responding to the questions above, please make an overall assessment of whether community ownership and participation to increase full supportive care for sick and small/LBW newborns is:**   - **Good** *(not a bottleneck to scale up)* - **Needs some improvements** *(minor bottleneck to scale up)* - **Needs major improvements** *(significant bottleneck to scale up)* - **Inadequate** *(very major bottleneck to scale up)* |

**IDENTIFICATION OF SOLUTIONS** **TO ADDRESS THE CHALLENGES**

*Please add sheets as appropriate*

| **Intervention 9: *INPATIENT SUPPORTIVE CARE FOR SICK AND SMALL NEWBORNS - Focus on IV fluids, feeding support, and safe oxygen*** | | |
| --- | --- | --- |
| **Summary of key bottlenecks *by order of priority*** | | Strategies and solutions to address identified challenges and bottlenecks |
| *Building block* | *Priority bottlenecks* |  |
| *Leadership and Governance* |  |  |
| *Health Finance* |  |  |
| *Health Workforce* |  |  |
| *Essential Medical products and Technologies* |  |  |
| *Health Service Delivery* |  |  |
| *Health Information Systems* |  |  |
| *Community Ownership and Participation* |  |  |
